# Sustainable strategies for remediation of Cr(VI) contaminated soil and water bodies: exploring the potential of invasive bamboo and its derivatives

**DOI:** 10.3389/fpls.2026.1753291

**Published:** 2026-03-23

**Authors:** Chirasmita Mohanty, Pratyush Kumar Das, Ramandeep Kaur, Chinnadurai Immanuel Selvaraj

**Affiliations:** 1 Department of Biotechnology, School of Bio Sciences and Technology (SBST), Vellore Institute of Technology, Vellore, Tamil Nadu, India; 2 Department of Phytopharmaceuticals, School of Agricultural and Bio-Engineering (SoABE), Centurion University of Technology and Management, Paralakhemundi, Odisha, India; 3 Department of Botany, Hans Raj Mahila Maha Vidyalaya, Jalandhar, Punjab, India; 4 Department of Genetics and Plant Breeding, VIT School of Agricultural Innovations and Advanced Learning (VAIAL), VIT, Vellore, Tamil Nadu, India

**Keywords:** adsorbent, bamboo, hexavalent chromium, phytoremediation, remediation, toxicity

## Abstract

Hexavalent chromium is a highly noxious and mobile environmental pollutant primarily released through industrial activities such as tanning, electroplating, mining, and pigment manufacturing. Cr(VI) exhibits mutagenic and carcinogenic properties due to its strong oxidizing nature, and poses severe risks to soil health, aquatic ecosystems, and living organisms. Its high solubility facilitates leaching through soil matrices, resulting in groundwater contamination and long-term ecological damage. Although conventional physicochemical remediation techniques are effective, their high operational; cost, energy demand, and generation of secondary pollutants limit their sustainability. The present review explores bamboo, and invasive and fast-growing plant with high biomass productivity, as a sustainable alternative for the remediation of Cr(VI) contaminated soil and water bodies. The phytoremediation potential of bamboo is discussed with emphasis on chromium uptake, immobilization, rhizospheric interactions, and tolerance mechanisms in contaminated soils. Furthermore, the application of bamboo-derived materials, including biochar, hydrochar, activated carbon, charcoal, and chemically modified bamboo-based adsorbents, is critically evaluated for Cr(VI) removal from aqueous systems through adsorption, reduction, and stabilization mechanisms. Comparative insights into the performance of bamboo-based adsorbents relative to conventional commercial materials are also presented. Finally, existing challenges related to large-scale application, material regeneration, and ecological risks associated with bamboo invasiveness are highlighted, and future research directions focusing on bamboo-based composite and recyclable materials are proposed to enhance remediation efficiency while minimizing secondary environmental impacts.

## Graphical Abstract

**Figure:**
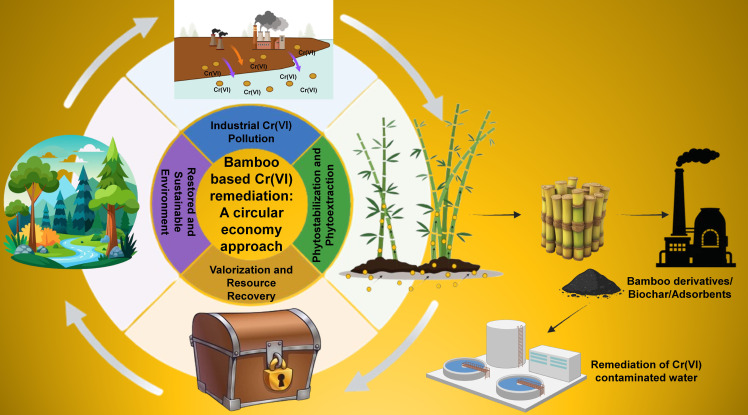

## 1 Introduction

Chromium is a transition metal naturally found in the Earth’s crust, but its amount in the environment is currently increasing significantly due to anthropogenic activities. The element occurs in two stable oxidation states: the relatively harmless, immobile trivalent chromium [Cr(III)] and the highly soluble, mobile, and toxic hexavalent chromium [Cr(VI)], which is predominant (Ray, 2017). Cr(VI) is commonly present in the form of the chromate anion (CrO_4_^2-^) or the dichromate anion (Cr2O_7_^2-^) in the environment. Its toxicity is attributed to its high oxidative potential, promoting penetration to biological membranes, thus causing carcinogenic, mutagenic, and teratogenic effects. Significant contributors to the Cr(VI) pollution of soil and water include major industrial processes like textile dyeing, leather tanning, electroplating, stainless steel manufacturing, and mining (Nur-E-Alam et al., 2020).

While considerable attention has been paid to Cr(VI) contamination in water bodies because of its high mobility, Cr(VI) pollution of soil remains a critical concern. Field surveys in industrially affected districts of eastern and central India have revealed that the uppermost soil layers exhibit elevated levels of Cr(VI), particularly in proximity to electroplating facilities, mining tailings, and tannery waste disposal sites (Choppala et al., 2013; Basu et al., 2024). The deposition of chromium−rich sludge and effluents onto terrestrial surfaces contributes to soil acidification, impairs microbial processes, and leads to a long−term reduction in soil fertility. Moreover, the weak sorption of Cr(VI) in diverse soil textures facilitates its leaching into underlying groundwater and subsequent uptake by crops, thereby posing a threat to the food chain and jeopardizing both ecological integrity and agricultural sustainability (Sharma et al., 2020).

In the major industrial provinces of India, Cr(VI) contamination is predominantly derived from effluents emitted by tannery, electroplating, textile, pulp−paper, and sugar−manufacturing sectors. The inadequate disposal of residues generated during chromite ore processing has caused severe groundwater contamination, with concentrations surpassing permissible limits, especially in the Chhattisgarh region (Banchhor et al., 2020). Industrial and municipal discharges have polluted agricultural soils and waters in Sonepat (Haryana) and along the Hindon River (Uttar Pradesh), endangering aquatic ecosystems and compromising food safety (Singh and Sharma, 2018; Rani et al., 2023a). Past mining operations, ship−breaking activities, and urban runoff have led to sediment contamination and ecological risks for aquatic biota in riverine systems such as Bhavnagar in Gujarat and the Zuari and Kushavati rivers in Goa (Rane and Matta, 2019; Gosai and Mankodi, 2024). Cr(VI) pollution in these regions threatens ecosystem health and poses significant risks to wildlife and human populations, underscoring the urgent requirement for comprehensive waste−management and remediation strategies (**Table 1****).**

**Table 1:** Major sources and ecological impact of Cr(VI) contamination across industrial regions in India.

Region/area	Major sources of Cr(VI)	Concentration levels	Ecological impact	Ref.
Hindon River, Uttar Pradesh	Industrial effluents (sugar, pulp, paper, tannery, textile, electroplating), municipal sewage	Up to 0.096 mg/L (summer), 0.088 mg/L (winter)	Severe water pollution, aquatic ecosystem toxicity, and potential carcinogenic risk to humans and animals	(Singh and Sharma, 2018)
Chhattisgarh	Chromite ore processing residue (COPR) disposal	Up to 1050 mg/L (leachate), 22 mg/L (surface water), 0.26 mg/L (groundwater)	Large-scale surface and groundwater contamination, acute health effects in cattle and residents	(Banchhor et al., 2020)
Bhavnagar Coast, Gujarat	Shipbreaking, municipal discharge, industrial operations	Mean Cr: 42 mg/kg in sediments	Minimal to moderate ecological risk in marine sediments; shipbreaking and urban runoff as key contributors	(Gosai and Mankodi, 2024)
Sonepat, Haryana	Industrialization (various industries)	Mean Cr: 44 mg/kg in agricultural soils	Moderate ecological risk; Cr among the top five heavy metals detected; possible health risk via the food chain	(Rani et al., 2023a)
Zuari & Kushavati Rivers, Goa	Past mining activities, industrial discharge	Elevated Cr in river sediments	Persistent sediment contamination, potential risk to aquatic life	(Rane and Matta, 2019)

The interaction of Cr(VI) with soil matrices plays a decisive role in determining the ultimate fate of Cr(VI), which is governed by a number of abiotic and biotic factors such as microbial activity, organic matter content (Wang et al., 2024), soil pH (Shi et al., 2023), and redox potential (Rasool et al., 2025). Under alkaline and oxidizing conditions, Cr(VI) resides in a mobile and soluble phase, thereby increasing the likelihood of leaching into groundwater, whereas Cr(III) is less soluble and readily precipitates onto soil particles. The transformation of Cr(VI) to Cr(III) occurs in reducing and acidic environments. However, the process is typically slow and may not be fully achieved in soils with low organic matter (Jiang et al., 2023). Moreover, Cr(III) can be oxidized back to Cr(VI) when the redox condition fluctuates, thereby complicating remediation efforts. This dynamic equilibrium between Cr(VI) and Cr(III) presents challenges for long-term stabilization and necessitates strategies aimed at ensuring permanent immobilization (Varadharajan et al., 2017).

Ion exchange adsorption, chemical reduction and precipitation, activated carbon, electrochemical treatment, phytoremediation, and microbial remediation constitute a spectrum of conventional remediation approaches employed to mitigate Cr(VI) contamination (**Table S1**). Ferrous sulfate or sodium metabisulfite are commonly used reagents for the chemical reduction of Cr(VI) to Cr(III); the subsequent precipitation step is quite effective, yet it is obstructed by high costs and the production of toxic sludge (Malaviya and Singh, 2016; Ahmad et al., 2021a; Kleinberg et al., 2023). Ion exchange resin−based adsorption is effective but requires frequent regeneration (Acharyya et al., 2023). Electrochemical techniques have demonstrated superior performance in removing Cr(VI); however, they are limited by infrastructure demands and high energy consumption, rendering them less suitable for rural settings. Pseudomonas and Bacillus species represent promising options for microbial remediation, yet their efficacy is constrained by environmental conditions that influence reaction kinetics and microbial viability (Ahmad et al., 2021a).

Phytoremediation has emerged as an ecologically sound alternative, presenting an economical and sustainable method for the remediation of sites contaminated with heavy metals. The invasive bamboo species prevalent in the Indian countryside possesses yet untapped potential for the remediation of chromium(VI)-contaminated sites (Saikia et al., 2024).

Bamboo distinguishes itself among various plant taxa due to its rapid growth rate, extensive and dense root system, substantial biomass yield, and tolerance to heavy-metal stress (Bian et al., 2023; Cao et al., 2025). In particular, *Phyllostachys pubescens* has demonstrated the capacity for both phytoextraction and phytostabilization of Cr(VI) (Ranieri et al., 2023). Empirical evidence indicates that bamboo predominantly accumulates chromium within its roots and rhizomes, thereby considerably reducing the risk of metal translocation to aboveground tissues and subsequent entry into the food chain. A pot experiment conducted in the Mediterranean region revealed that Moso bamboo can accumulate 3.9 mg g^-^¹ dry weight of chromium in its roots over 84 days, with only minimal translocation to the shoots (Ranieri et al., 2020). This capability of bamboo to tolerate and immobilize Cr(VI) renders it a preferred candidate for remediation processes in India.

In addition to its phytoremediation potential, bamboo is regarded as a raw material for carbonaceous derivatives and biochar production. Bamboo-derived biochar has been extensively investigated due to its advantageous properties, including a large surface area, substantial porosity, and the presence of functional groups that play a pivotal role in the adsorption and reduction of Cr(VI). Numerous modifications have been introduced to optimize performance. For example, bamboo-derived biochar activated with potassium bicarbonate (KHCO_3_) and doped with nitrogen has exhibited remarkable Cr(VI) adsorption capacity, reaching up to 385.8 mg g^-^¹ under acidic conditions (Mi and Wang, 2024). Furthermore, chitosan-functionalized magnetic bamboo biochar has demonstrated significant efficacy, achieving a removal capacity of 121.8 mg g^-^¹ while retaining recyclability over multiple cycles (Yu et al., 2023). The incorporation of nanoscale zero-valent iron (nZVI) into bamboo-derived biochar promotes the reduction of Cr(VI) to Cr(III) via electrostatic attraction and surface complexation. These modified derivatives function through diverse mechanisms, involving redox transformation, ion exchange, electrostatic attraction, and surface complexation, thereby rendering them highly effective for the remediation of contaminated water and soil (Rychluk et al., 2024).

The mechanisms by which bamboo and its derivatives facilitate the remediation of Cr(VI) are multifaceted, comprising root uptake, rhizosphere adsorption, and the enhancement of microbial consortia that facilitate phytoremediation. The metabolites and organic acids released by the extensive root network chelate chromium, thereby increasing its solubility and facilitating uptake (Mi and Wang, 2024). Cr(VI) anions are adsorbed onto the root surface through electrostatic interaction and complexation with hydroxyl and carboxyl functional groups (Kumar et al., 2016). Rhizobacterial species, including Bacillus and Pseudomonas, can enzymatically convert Cr(VI) to Cr(III), further augmenting detoxification processes (Guo et al., 2021). The biochar’s adsorption capacity is influenced by multiple physicochemical attributes, including surface area, pore volume, and active functional groups. Electron donation initiates reductive transformations from surface-bound Fe(II) or carbon, culminating in the formation of Cr(III) hydroxide within the biochar matrix (Wang et al., 2023). Bamboo and its products combine both natural and engineered approaches to provide a good strategy that can be used to remediate Cr(VI) effectively and sustainably. The method reduces the ecological effects of Cr(VI) and, at the same time, helps to leverage the use of invasive bamboo species, making it difficult to have significant ecological control. When considered through the circular economy, bamboo can grow on polluted ground. It can be harvested to yield biomass and then turned into biochar that can be used to remediate the soil (Rani et al., 2023a). Bamboo-based alternatives to the existing systems have cost-effectiveness, scalability, and multifunctional properties, so they can be considered a reasonable choice in the developing setting, such as India, where industrial contamination and resource scarcity often collide (Kaur et al., 2022). This closed-loop approach is suitable in terms of sustainability goals and is responsive to the increasing need to have low-cost remediation technologies in India.

## 2 Cr(VI) as an environmental pollutant

**Figure 1:**
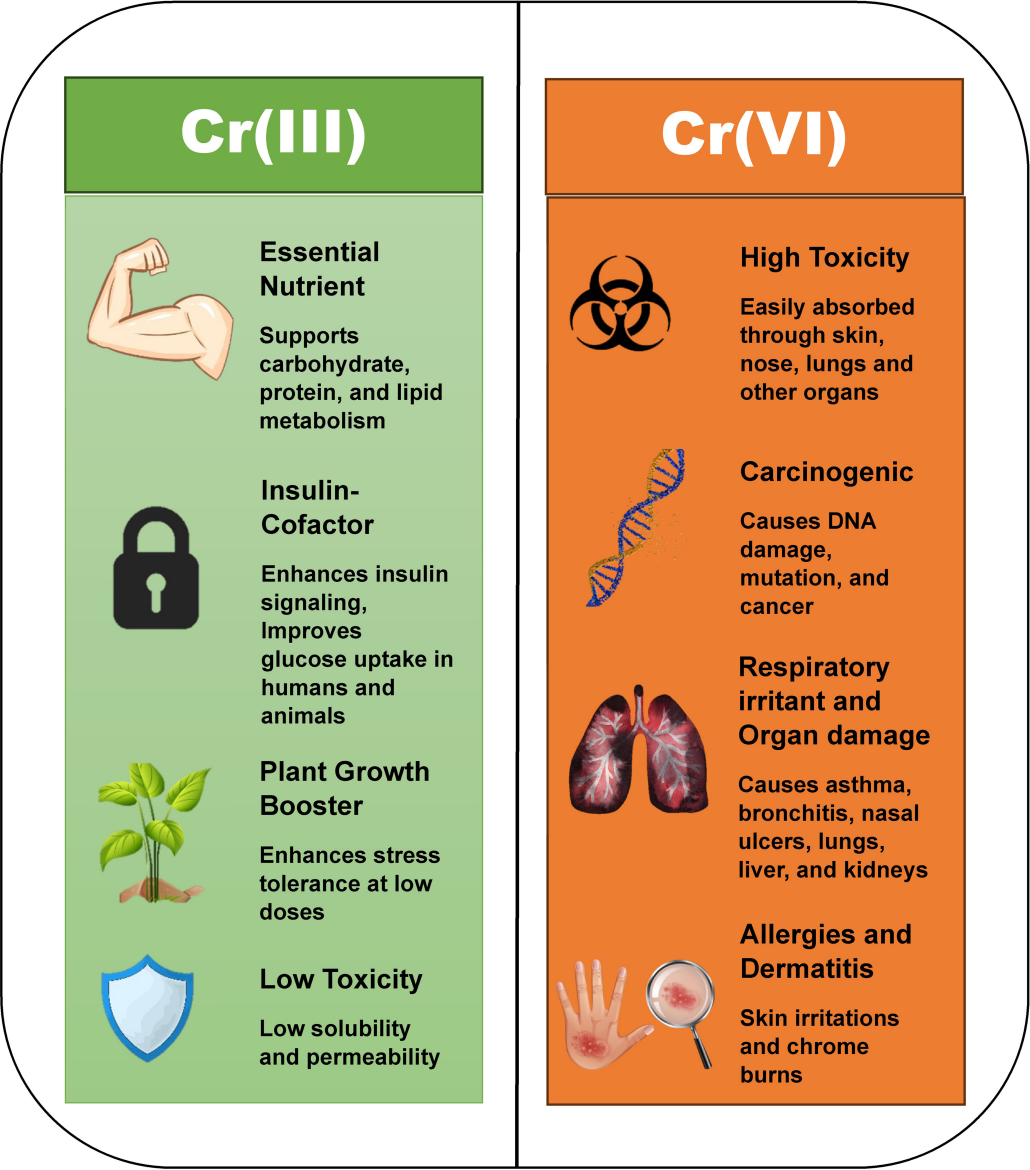
Comparative account of the benefits of Cr(III) and the harmful effects of Cr(VI).

Chromium (Cr) is a relatively abundant transition metal that has been extensively employed across diverse industrial contexts. It belongs to the d−block element of group 6 within the periodic table, exhibiting several characteristic physical properties, including a melting point of 1907°C and a boiling point of 2672°C, a pronounced hardness, metallic lustre, and intrinsic anticorrosive behavior, thereby rendering it among the most valuable feedstocks for large−scale manufacturing processes (Pradhan et al., 2017). In comparison to other heavy metals, Chromium is particularly noteworthy for its dualistic character, functioning simultaneously as a biologically essential micronutrient and, when present in excess or in inappropriate oxidation states, as a hazardous environmental contaminant. The usefulness and toxicity of this heavy metal are governed by its oxidation state. The trivalent or Cr(III) and the hexavalent or Cr(VI) are the two most prevalent forms of Cr in the environment. Cr(III) has been found to serve as a micronutrient in humans, animals, and plants. Children up to six months of age are required to intake a daily dose of 10-40 µg of Cr(III), while the concentration varies between 25-35 µg for other age groups (Genchi et al., 2021). Cr(III) at required concentrations is quite beneficial for living organisms. Cr(VI) on the other side, is highly toxic even at low concentrations, thus leading to several harmful effects on the biotic components. **Figure 1** provides a comparative account of the benefits of Cr(III) and the detrimental effects of Cr(VI) on living biota.

### 2.1 Sources of Cr(VI)

Unlike Cr(III), which is naturally present, Cr(VI) is mostly produced from anthropogenic activities and released into the environment. Mining is one of the most common contributing factors. It is also aided by other industrial activities like leather tanning, metal welding, textile dyeing, printing, and the production of ceramics, cement, chemicals, pigments, and steel (**Table 2**). The pollution of soil, surface, and groundwater is mainly caused due to leaching of chromium-contaminated industrial wastes (Das et al., 2024).

**Table 2:** Production of Cr(VI) from different sources and their respective mode of exposure.

Industrial activity	Form	Mode of exposure	Affected matrices
Mining	Chromite	Mine wash water, Dust, and Particulate matter	Soil and Water
Leather tanning	Basic chromic sulfate, Chromic hydroxide, Ammonium dichromate, Sodium chromate	Effluent, Sludge	Soil and Water
Textile dyeing	Chromic acetate, Chromic chloride, Potassium chromate, Ammonium dichromate, Sodium dichromate	Effluent, Sludge	Soil and Water
Metal welding	Dichromium trioxide, Chromium trioxide	Fumes, Effluent	Air, Soil, and Water
Printing	Chromic acetate, Chromic nitrate	Effluent, Sludge	Soil and Water
Chrome plating	Chromium trioxide	Effluent	Soil and Water
Cement manufacturing	Calcium chromate	Effluent	Air, Soil, and Water
Steel manufacturing	Ferrochromium	Effluent	Soil and Water
Chemical manufacturing	Potassium dichromate	Effluent	Soil and Water
Pigment manufacturing	Chromic oxide, Chromium trioxide, Potassium dichromate, Sodium chromate, Lead chromate	Effluent	Soil and Water

Source: (International Agency for Research on Cancer, 1990).

### 2.2 Global scenario of Cr(VI) contamination in water bodies

Global concern about the increasing risk of Cr(VI) pollution in water bodies is gaining significant traction due to its widespread use and emissions resulting from anthropogenic activities. Cr and Cr(VI) discharge limits are regulated nationally and vary from nation to nation (Brasili et al., 2020). Much lesser as compared to anthropogenic activities, natural processes like weathering of rocks also contribute towards Cr(VI) contamination of soil and water bodies. Cr(VI) contamination of water sources has been widely observed in the European Union (EU). 512 installations from the EU have been registered under the European Pollutant Release Register due to the release of high concentrations of Cr into the air and water (Tumolo et al., 2020). The majority of these facilities mostly belong to the energy sector (699.94 tons, 83.9%) followed by waste and wastewater management (90.99 tons, 10.9%), metal processing and production (18.50 tons, 2.2%), chemical manufacturing (17.89 tons, 2.1%), minerals (3.99 tons, 0.5%), and paper and wood production (2.43 tons, 0.3%). Individual member states of the EU have their own regulations to monitor Cr emissions in the aquatic environment (Vaiopoulou and Gikas, 2020). Some countries in the union, like Austria, Denmark, and Greece, only regulate the total chromium concentration in the water, while some other nations regulate Cr and Cr(VI) separately (Pradhan et al., 2017; Bharagava and Mishra, 2018). Among the EU nations, Germany, Poland, Italy, and the UK were the major Cr emitters, followed by others (**Supplementary Figure 1**).

Besides the EU, some other parts of the world also contribute to high levels of aquatic Cr(VI) pollution, including China and India. China is the largest producer of Cr slag, with numerous facilities producing different Cr salts, resulting in an annual discharge of 450,000 tons. Qujing in Yunnan province of China witnessed a serious environmental issue in 2011 when Lvliang Chemical Industry Co., Ltd. illegally dumped almost 5000 tons of untreated Cr slag into Nanpan River, thus raising the heavy metal concentration in the river water to 2000 times more than the permissible standards. This further led to the death of 7 villagers due to cancer. Approximately 77 numbers of cattle and sheep suddenly died due to the consumption of the severely polluted river water (Gao and Xia, 2011).

### 2.3 Fate of Cr(VI) in water bodies

Chromium can be found in two stable forms in aquatic ecosystems: Cr(III) and Cr(VI). The redox chemical characteristics of Cr(VI) influence its behavior in aquatic environments. Cr can undergo either oxidation or reduction, thereby leading to the formation of intermediates and thus governing the type of treatment processes to be undertaken. The **Supplementary Table 2** provides an account of the predominant chemical species of Cr at environmental pH conditions ranging between 5–9 in the aquatic environment.

#### 2.3.1 Oxidation of Cr

**Figure 2:**
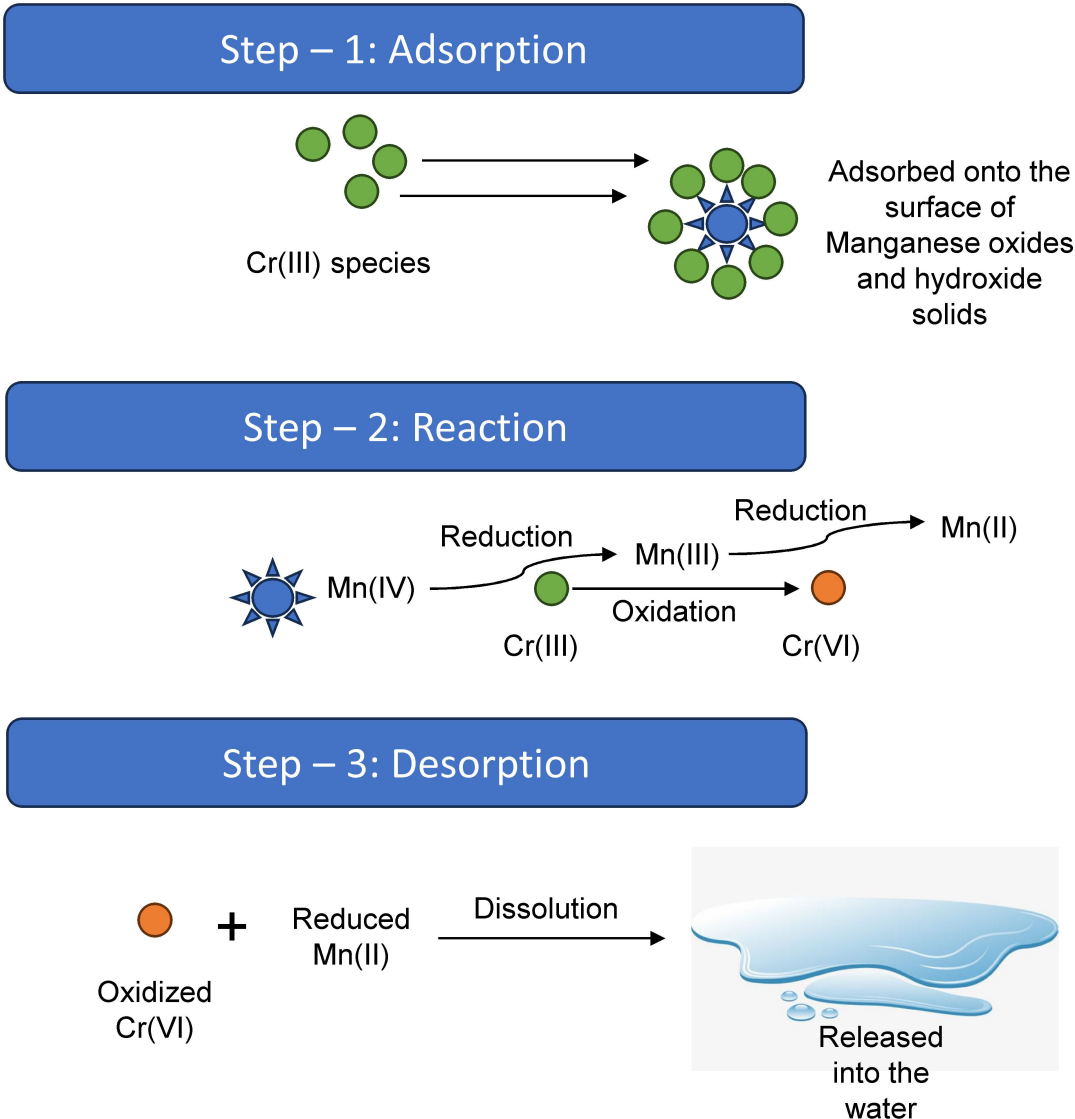
The oxidation of Cr(III) to Cr(VI) is a three-step process. (Step – 1: Adsorption): In this step, the Cr(III) gets adsorbed onto the surface of Manganese oxides and hydroxides solids. (Step – 2: Reaction): In this step, the Mn(IV) present on the solid surface gets reduced to Mn(III), which is a strong oxidant, thereby oxidizing Cr(III) to Cr(VI). (Step – 3: Desorption): In this step, the reduced Mn(II), along with the Cr(VI), are dissolved and released in water.

In an aquatic environment, proximal to the water-sediment interface, several oxidants lead to the oxidation of Cr. The oxidation process mostly involves the conversion of Cr(III) into Cr(VI) under the influence of several parameters, like dissolved oxygen, manganese hydroxides, and bacterial activity. The aerobic oxidation of Cr(III) to Cr(VI) is practically possible in aquatic environments; however, the process is very slow. Aerobic oxidation only occurs under drastic conditions, mostly at high temperatures (200°C - 300°C) in the aquatic environment, which acts as a limiting factor (Apte et al., 2006). Oxidation of Cr(III) to Cr(VI) is also facilitated by the hydroxides of Mn(III) and Mn(IV). The oxidation process is largely dependent on the different species of these hydroxides, which may be further attributed to their crystallinity, valence, and surface charge. The oxidation of Cr(III) to Cr(VI) via Mn hydroxides follows certain steps as depicted in **Figure 2**.

Oxides of manganese also play a major role in the oxidation of Cr(III) to Cr(VI). Reduction of Mn hydroxides under anoxic conditions in aquatic bodies leads to the formation of Mn^2+^ precipitates (Ao et al., 2022). The reoxidation of the Mn^2+^ anoxic sediments to Mn(III/IV) hydroxides due to events like flooding, sailing of boats, and oxidation at a pH of 7 results in the oxidation of Cr(III) to Cr(VI) (Ao et al., 2024).

Bacterial activity is also responsible for the oxidation of Mn(II) to hydroxides of Mn(IV) via two enzyme-based electron transfer reactions, with Mn(III) being produced as an intermediate. This intermediary product acts as a strong oxidant of Cr(III), leading to the generation of the toxic Cr(VI) (Tu et al., 2025).

#### 2.3.2 Reduction of Cr

Cr(VI) is efficiently converted to Cr(III) by Fe^2+^, HS^-^, organic matter, and bacteria. In water, dissolved Fe^2+^ reduces Cr(VI) to Cr(III) via three successive electron transfer systems. The kinetics of Cr(VI) reduction increase with the rise pH from 5 to 9, thus increasing the production of Cr(III) (Gorny et al., 2016). The interface of water and sediments, dissolved oxygen oxidizes Fe^2+^ to Fe^3+^, thereby reducing Cr(VI) (Morgan and Lahav, 2007). Sulfides dissolved in water are also responsible for the reduction of Cr(VI) to Cr(III) through a three-step electron transfer process. The production of elemental sulfur under anaerobic conditions during the reduction of Cr(VI) has been found to increase Cr(III) production (Lan et al., 2005). The reduction of Cr(VI) to Cr(III) is also facilitated by particulate organic matter (electron donor). The reaction kinetics of Cr(VI) reduction through particulate organic matter is, however quite low when compared to the reduction process facilitated by Fe^2+^ or S^2-^ (Wittbrodt and Palmer, 1996). Cr(VI) reduction is also facilitated by certain microbes as a kind of defense mechanism under both aerobic and anaerobic conditions. Aerobic reduction typically occurs through chromate reductase enzymes or electron donors available during metabolic processes, while anaerobic reduction often involves Cr(VI) serving as a terminal electron acceptor in microbial respiration. The **Supplementary Table 3** summarizes representative microbes reported to reduce Cr(VI) under different redox regimes. There are several factors that affect the Cr(VI) reduction ability of bacteria, including concentration of bacterial biomass, Cr(VI) concentration in the aquatic environment, presence of nutrient sources like carbon, pH, temperature, and redox potential (Chen and Hao, 1998). The rate of reduction of Cr(VI) via microbes is higher in aerobic environments, while under anaerobic conditions, Fe^2+^ serves as the primary reductant at pH>5, and sulfides predominate under more acidic conditions (pH<5) (Fendorf et al., 2000).

#### 2.3.3 Precipitation and adsorption

Cr(VI) also undergoes certain other reactions in the aquatic environment, including precipitation and adsorption. Cr(VI) is highly soluble but forms limited precipitates by getting incorporated into solid matrices by substituting sulfides and carbonates (Kimbrough et al., 1999). Precipitation generally results in a decrease in the solubility of Cr(VI) in the sediment pores in water bodies. The oxidation state of Cr governs the adsorption process. The process of adsorption of Cr(VI) differs according to the surface of the minerals. Adsorption of Cr(VI) on aluminum hydroxide surfaces involves electrostatic interactions, while iron hydroxide surfaces involve stronger covalent binding (Ajouyed et al., 2010). Surface sediments in aquatic systems containing amorphous minerals with positively charged sites enhance the adsorption of anionic Cr(VI) species. The sorption of Cr(VI) rises with a fall of pH and vice versa. The type of minerals also governs the sorption of Cr(VI) to some extent, with iron hydroxides having the highest sorption ability, followed by aluminum oxide, kaolinite, and montmorillonite. The aquatic environment also contains several anionic or neutral species with concentrations in the µM to mM range that compete with Cr(VI) to bind at the positive sites of the minerals. These species generally contribute towards a decrease in Cr(VI) sorption and an increase in mobility of the toxic heavy metal in the sediments (Gorny et al., 2016). Particulate organic matter forms the second most important adsorbent in aquatic sediments. Sorption of Cr(VI) onto this particulate matter generally follows two mechanisms. One of the mechanisms is the indirect sorption through a cationic bridge formed between the Cr(VI) anion and the negative charge on the surface of the particulate matter. The other mechanism involves a direct process wherein the sorption of anionic Cr(VI) occurs at positively charged groups on the surfaces of the organic solid phase (Park et al., 2008).

### 2.4 Fate of Cr(VI) in soil

The behavior of Cr(VI) in soil is complex and influenced by various abiotic and biotic factors (**Table 3**). When introduced into terrestrial ecosystems via industrial discharges, sludge disposal, or atmospheric deposition, Cr(VI) persists in a mobile and bioavailable state under typical surface soil conditions, particularly in alkaline or oxidizing environments. The mobility of chromium poses significant concerns due to its potential to contaminate groundwater and accumulate in plant tissues (Khatua and Kumar Dey, 2023). The present investigation has demonstrated that soils proximal to tannery and chromite mining operations exhibit elevated concentrations of Cr(VI), underscoring the considerable environmental hazards associated with these industrial activities. Surface runoff and the direct application of chromium−laden waste further aggravate soil contamination, with the most pronounced effects observed in the Indian states of Odisha and Tamil Nadu (Tadesse and Guya, 2017; Das et al., 2021).

**Table 3:** Key soil factors influencing chromium(VI) mobility and transformation.

Soil factor	Influence on Cr(VI) behavior	Remarks
pH	Alkaline pH increases its solubility, hence promotes the mobility of Cr(VI), whereas acidic conditions favor the reduction of Cr(VI) to the less mobile Cr(III) form.	The presence of hydroxyl groups in soil minerals can facilitate the adsorption of Cr(VI), affecting its mobility and bioavailability (Pattnaik and Sahu, 2022).
Redox potential	Oxidizing conditions maintain mobile form Cr(VI), while reducing conditions favor the formation of more stable Cr(III).	The redox potential is crucial for the long-term immobilization of chromium in soils (Amanatidou, 2023).
Organic matter	Organic matter enhances microbial activity and provides reducing agents that promote the reduction of Cr(VI) to Cr(III).	Soils with higher organic content can effectively reduce Cr(VI), promoting natural attenuation (Zhang et al., 2022).
Microbial activity	Microbial activity plays a significant role in the enzymatic reduction of Cr(VI) to Cr(III), although it is sensitive to Cr toxicity and soil quality.	Certain bacteria, algae, and fungi can be utilized for bioremediation to address elevated Cr levels in soils (Pattnaik and Sahu, 2022).
Presence of oxidants	Manganese oxides are key oxidants that can reoxidize Cr(III) back to Cr(VI), influencing chromium dynamics in soils	The presence of Mn oxides in soils is directly related to the oxidation potential of chromium (Hao et al., 2022).

Cr(VI) exhibits high aqueous solubility, predominantly existing as chromate (CrO_4_^2−^) and dichromate (Cr_2_O_7_^2−^) ions, which display weak sorption affinity for clay minerals and humus. Consequently, the risk of vertical migration into subsoil layers and aquifers is increased (Wang et al., 2020a). In reducing environments, such as waterlogged soils or those rich in organic matter, Cr(VI) is reduced to the more stable and less toxic Cr(III). This species preferentially precipitates as Cr(OH)_3_ or associates strongly with soil particles. In alkaline soils, Cr(VI) traverses pore water as the chromate anion(CrO_4_^2-^), whereas Cr(III) is predominantly present as amorphous Cr(OH)_3_ and other poorly soluble precipitates (Zhu et al., 2023).

Specific soil-dwelling microbial assemblages, including bacteria and fungi, play a key role in chromium biotransformation owing to their enzymatic systems that facilitate the reduction of Cr(VI) to Cr(III). Nonetheless, elevated concentrations of Cr(VI) can exert deleterious effects on these microbial communities, perturbing the soil’s ecological equilibrium and diminishing its intrinsic detoxification capacity (Arishi and Mashhour, 2021). For instance, studies investigating microbial respiration rates in chromium−contaminated soils surrounding Sukinda, Odisha, revealed a marked reduction in aerobic microbial activity relative to uncontaminated sites. Certain bacterial taxa, such as Bacillus spp. and Enterobacter cloacae, have demonstrated pronounced resistance to Cr(VI) and efficiently convert it to Cr(III) (Harish et al., 2012; Das et al., 2024). Alterations in microbial community composition further constrain biotransformation potential, thereby rendering bioremediation efforts more challenging.

Moreover, the redox cycling of chromium represents a significant obstacle to remediation. Cr(III) can be oxidized back to Cr(VI) in the presence of potent oxidizing agents under variable moisture and temperature regimes (Hao et al., 2022). Effective remediation strategies must therefore not only promote the reduction of Cr(VI) but also ensure its stabilization in a non−toxic form. The persistent presence of Cr(VI) imposes considerable risks to biodiversity, agriculture, and groundwater resources.

### 2.5 Cr(VI) led environmental toxicity

Cr(VI) is a highly toxic form of heavy metal that poses carcinogenic properties and is also considered a neurotoxicant (Wise et al., 2022). Cr(VI) toxicity affects both plants and animals, including humans. Cr(VI) enters the human body and cells via certain distinct routes, including oral, nasal, and dermal. An exposure to Cr(VI) for a short period of 14 days is referred to as acute, while exposure for 365 days or more is considered chronic. Any exposure between 75–364 days is intermediate (Yang et al., 2020). Cr(VI) exposure mostly leads to skin and nasal irritation, ulceration, allergies, dermatitis, bronchitis, asthma, DNA damage, and cancer (Vendruscolo et al., 2017). The rapid uptake of Cr(VI) into human and animal cells is mostly mediated through the sulfate carriers. Once inside the cell, the Cr(VI) gets reduced to Cr(III) and, in the process, leads to the formation of several intermediary species like Cr(IV) and Cr(V), along with the generation of reactive oxygen species (ROS). These reactive oxygen species and other intermediates are the main culprits behind human lung carcinogenesis (Das et al., 2021).

Cr(VI) is recognized as a hazardous contaminant on a global scale owing to its high solubility and mobility, which facilitate widespread biological damage. Cr(VI) is not required for biological processes and is instead associated with mutagenic and carcinogenic effects, whereas its trivalent counterpart, Cr(III), possesses essential metabolic roles. Its analogous structure to sulfate and phosphate ions permits Cr(VI) to traverse cellular membranes via conventional transport channels, thereby entering cells and initiating a cascade of oxidative stress and subsequent cellular injury (Zhigalenok et al., 2025).

The solubility of Cr(VI) permits swift absorption and accumulation across a diverse array of organisms, including plants, microbes, aquatic species, and terrestrial animals (**Table 4**). Its presence disrupts critical soil enzymes, hampers plant development, diminishes yields, and compromises aquatic biodiversity by affecting fish populations and other organisms. Cr(VI), even at trace concentrations, can induce DNA damage in aquatic species, inhibit plant germination, and suppress beneficial microbial activity, culminating in extensive ecological degradation (Yan et al., 2023).

**Table 4:** Cr(VI)-induced toxicity across different biological domains.

Affected system	Major effects	Mechanism	Regional evidence
Humans	Microcytic anemia, mitochondrial dysfunction and genotoxic injury in blood cells, leading to cancer, occupational asthma, airway hyper-sensitivity, and irritation of nose, eye, and skin.	Oxidative stress, DNA adducts, GSH depletion	Sukinda Valley, Odisha (Mohanty et al., 2023)
Animals	Liver/kidney damage, growth inhibition, reproductive dysfunction, and Neurotoxicity.	Bioaccumulation, ROS generation, apoptosis	Livestock near tanneries (Kapoor et al., 2022)
Plants	Reduced germination rate, stunted growth, chlorosis, and root toxicity	Nutrient uptake inhibition, oxidative damage	Sukinda Valley, Odisha (Mohanty et al., 2023)
Microbes	Reduced biomass and enzyme activity, nitrification inhibition	Membrane disruption, DNA oxidation	Cr(VI) polluted regions (Tang et al., 2021)

#### 2.5.1 Toxicity in humans

Humans can be exposed to Cr(VI) through inhalation, ingestion of contaminated food or water, and dermal contact, particularly in occupational settings such as leather tanning, electroplating, and chromite mining (Yatera et al., 2018). Chronic exposure is associated with a range of adverse health effects, including nasal septum perforation, dermatitis, nephrotoxicity, respiratory diseases, and various cancers, most notably pulmonary carcinoma (Shin et al., 2023). Cr(VI) functions as a potent skin irritant and sensitizer; it can provoke respiratory diseases when inhaled, cause gastrointestinal irritation and disorders, exert neurotoxic effects, damage renal and hepatic tissues, and pose reproductive toxicity (**Table 5**). Cr(VI) enters cells via anionic transporters and is subsequently reduced to Cr(III), generating reactive oxygen species (ROS). This provoke oxidative stress, DNA damage, apoptosis, inflammatory responses, endoplasmic reticulum stress, autophagy, and pyroptosis, which together contribute to its carcinogenic potential (Zhitkovich, 2011). Epidemiological studies in industrial regions of India, including Kanpur, and Sukinda Valley, reported elevated chromium levels in blood and urine, along with skin lesions, gastrointestinal issues, and respiratory diseases among exposed workers (Choudhari et al., 2021; Mohanty et al., 2023). Studies demonstrate that prenatal exposure is deleterious; maternal ingestion of Cr(VI)-contaminated water can lead to low birth weight, developmental delays, and skeletal anomalies in infants (Putra et al., 2024).

**Table 5:** Systemic toxicity and mechanisms of Cr(VI) exposure.

System affected	Exposure route	Toxic effects	Mechanism of toxicity	Ref.
Integumentary (Skin)	Dermal contact	Sensitization, irritation, allergic contact dermatitis, ulceration	Type IV hypersensitivity, inflammation, and DNA repair inhibition	(Reif and Murray, 2024)
Respiratory System	Inhalation (occupational)	Asthma, Nasal septum perforation, Lung irritation, pulmonary fibrosis, and lung cancer	DNA damage, oxidative stress, inflammation	(Reif and Murray, 2024)
Gastrointestinal	Ingestion	Mucosal burns, Abdominal pain, inflammation, liver dysfunction	Oxidative stress, apoptosis, liver impairment	(Sun et al., 2015; Singh et al., 2022)
Central and peripheral Nervous System	Inhalation, systemic	Neurodegeneration, cognitive impairment	Oxidative stress, acetylcholinesterase dysfunction	(Reif and Murray, 2024)
Renal (Kidney)	Systemic (ingestion/ inhalation)	Acute tubular injury, chronic renal failure risk	Oxidative damage, mitochondrial impairment	(Reif and Murray, 2024)
Reproductive System	Systemic (ingestion/ inhalation)	Sperm damage, fetal toxicity	Oxidative stress, ROS-mediated DNA fragmentation leads to reproductive toxicity, apoptosis	(Shin et al., 2023)
Immune System	Systemic (Inhalation/ Oral)	Immune modulation, leukocyte alterations, hypersensitivity	Inflammatory cytokine release, immune suppression	(Shin et al., 2023)
Multiple Systems	Occupational/environmental exposure	Increased cancer risk (lung, nasal, bladder, stomach)	Genotoxicity, tumor promotion	(Wise et al., 2022)

#### 2.5.2 Toxicity in animals

Cr(VI) is a highly toxic environmental pollutant that adversely affects a wide range of terrestrial and aquatic animals. Exposure occurs via ingestion, inhalation, or dermal absorption and can result in bioaccumulation, systemic toxicity, and metabolic disruption. The underlying mechanisms involve cellular uptake of Cr(VI), intracellular reduction to Cr(III), generation of reactive oxygen species (ROS), oxidative damage to DNA, lipids, and proteins, mitochondrial dysfunction, endoplasmic reticulum stress, apoptosis, and autophagy, alongside immune suppression immune suppression (Sun et al., 2015; Machado et al., 2019).

Aquatic organisms, such as fish and amphibians, are especially susceptible due to the high solubility and mobility of Cr(VI) in water. Prolonged exposure to Cr(VI) in *Danio rerio* (Zebrafish) results in reproductive toxicity, including gonadal damage, hormonal imbalance, and offspring deformities), neurotoxicity such as brain injury, decreased AChE activity, and systemic oxidative stress (Zhao et al., 2024).

In terrestrial animals, Cr(VI) induces liver and kidney damage, altered metabolic pathways, and carcinogenicity (**Table 6**). For instance, oral Cr(VI) exposure in broiler chicken caused significant hepatic cord disruption, diminished growth, and imbalances in amino acid and phenylalanine metabolism (Zhuo et al., 2023). Chronic ingestion of Cr(VI) in rodents led to increased occurrence of cancers, including oral squamous cell carcinoma and intestinal tumors, and induces hematological abnormalities, reproductive impairments, and mitochondrial dysfunction (Singh et al., 2022; Islam et al., 2024). In the Indian context, chromium accumulation in domestic animals near contaminated sites has been associated with reproductive failures and increased cancer incidence in livestock.

**Table 6:** Mechanisms and effect of Chromium (VI) exposure in various groups of animals.

Species	Exposure route	Observed effects	Mechanism of action	Ref.
Rats	Oral	Hepatotoxicity, nephrotoxicity, decreased sperm count	Lipid peroxidation, apoptosis	(National Toxicology Program, 2008; Hassan et al., 2019; US EPA, 2024)
Mice	Oral	Tumor formation, oxidative stress	DNA adduct formation, mitochondrial dysfunction	(National Toxicology Program, 2008; Islam et al., 2024)
Fish	Aquatic (waterborne)	Gill damage, behavioral changes	Oxidative stress in gills and brain	(Velma and Tchounwou, 2010)
Poultry	Oral via feed/water	Reduced growth, immune dysfunction	Oxidative damage, enzyme inhibition	(Zhuo et al., 2023)
Dogs	Oral	Oxidative heart damage	Oxidative stress, ATPase activity alteration	(Lu et al., 2019)

#### 2.5.3 Toxicity in plants

Cr(VI) toxicity poses a serious threat to plants due to its high mobility, solubility, and oxidative potential, causing serious physiological, biochemical, and morphological disruptions (Zulfiqar et al., 2023) (**Table 7**). The ion is taken up by roots through ion channels and rapidly accumulates in root cells, where it generates ROS. These ROS induce oxidative stress, damaging cellular components, disrupting cell division, and causing mutations and chromosomal abnormalities (Sharma et al., 2020).

**Table 7:** Mechanisms and effect of chromium (VI) exposure in different plants.

Species	Exposure route	Observed effects	Mechanism of action	Ref.
Pea (*Pisum sativum*)	Soil/root uptake	Dose-dependent inhibition of root and shoot growth	ROS generation, enzyme inhibition, oxidative damage	(Stambulska et al., 2018)
Legumes (General)	Soil/root uptake	Suppressed germination, reduced nodule function	Elevated ROS, pigment inhibition, and modification of proteins	(Stambulska et al., 2018)
Wheat (*Triticum aestivum*)	Soil/root uptake	Inhibited root elongation and shoot biomass	Inhibition of cell division, oxidative stress	(Datta et al., 2011)
Cluster bean (*Cyamopsis tetragonoloba*)	Soil/root uptake	Impaired enzyme activity, pigment loss, and growth decline	Inactivation of metabolic enzymes, ROS production	(Sangwan et al., 2014)
*Echinochloa colona*	Soil/root uptake	25% reduction in seed germination	Protease activation, amylase inhibition	(Rout et al., 2000)
Cotton	Soil; root uptake	Reduced height, biomass, and leaf area	Oxidative stress damages photosynthetic pigments	(Farooq et al., 2016)

Cr(VI) drastically lowers seed germination rates in species like cowpea, melon, and wheat, often resulting in delayed germination or seed mortality. Root development is severely affected, with aberrant thickening, decreased lateral roots and root hairs, reduced length, and poor secondary development. Shoots and leaves are also affected, with reductions in leaf area, biomass, and photosynthetic capacity, induction of chlorosis (yellowing) and necrosis (death of tissue), and suppressed chlorophyll synthesis. Plant height and flower or fruit size decline with increased Cr(VI) concentration (Saud et al., 2022).

Cr(VI) interferes with water uptake and transport, leading to decreased turgor, plasmolysis, and altered physiological water delivery in leaves. Structural alteration of the root cell wall diminishes the effectiveness of water and nutrient transport, resulting in stunted growth and decreased agricultural output (Sharma et al., 2023b). Photosynthesis is substantially impeded in spinach and pea species due to electron transport disruption in chloroplasts and reduced CO_2_ assimilation. Cr(VI) affects nitrogen absorption and general metabolism by interfering with essential metabolic enzymes such as glutamine synthetase, nitrogenase, and nitrate reductase. Prolonged exposure impairs the plant’s defense response and causes irreversible damage by reducing the activity of antioxidant enzymes at high Cr(VI) levels (Singh et al., 2022).

#### 2.5.4 Toxicity in microbes

Cr(VI) is a noxious heavy metal discharged into the environment from industrial operations such as tanning, electroplating, and manufacturing. Its strong oxidative nature leads to the generation of ROS, which damages cellular components including DNA, proteins, and lipids disrupt metabolic functions, and ultimately cause microbial cell death (Dubey et al., 2024; Ma et al., 2024c). Cr(VI) enters microbial cells via sulfate or phosphate transport channels due to its structural similarity to these essential ions, intensifying its toxic effects.

Exposure to Cr(VI) alters microbial community structure and reduces biodiversity in contaminated environments. It negatively impacts plant growth promoting traits of rhizospheric bacteria, such as the production of indole acetic acid, siderophores, and phosphate-solubilizing enzymes, even when microbial growth is not fully inhibited under sublethal concentrations (Majhi and Sikdar, 2025). High Cr(VI) concentrations further suppress metabolic activity, bioaccumulation capacity, and nutrient-cycling functions, compromising soil health.

Microbial species differ in their sensitivity to Cr(VI), where some bacteria undergo growth inhibition, cell cycle arrest, and oxidative damage, while others evolve resistance through mechanisms like enzymatic reduction of Cr(VI) to Cr(III) (**Table 8**). However, high levels of Cr(VI) typically cause reduction in the metabolic activity and bioaccumulation capacity. Overall, Cr(VI) contamination poses a dual threat by directly harming microbial populations important for ecosystem functioning and indirectly by impairing beneficial microbial activity in agricultural soil.

**Table 8:** Mechanisms and effects of chromium (VI) exposure in various microbial species.

Species	Exposure route	Observed effects	Mechanism of action	Ref.
*Pseudomonas* species	Direct water/soil exposure	Reduced plant growth-promoting traits (IAA, siderophores), growth inhibition at high Cr(VI)	Oxidative stress, enzyme inhibition, DNA damage, impaired nutrient cycling	(Kumar et al., 2020)
*Euglena* species	Aqueous exposure	Growth phase delay, cell cycle arrest, photosynthesis, and respiration inhibition	ROS generation, mitochondrial, and cytoskeleton damage	(Cervantes et al., 2001)
*Saccharomyces* species	Culture media exposure	Mitochondrial damage, oxygen uptake inhibition, and increased mutation rate	Chromate targets mitochondria, DNA strand breaks, and mutation induction	(Cervantes et al., 2001)
Soil microbial community	Soil contamination	Decreased microbial diversity, community shift	Toxicity via oxidative stress, metabolic disruption, Cr(VI) reduction inefficiency	(Sheik et al., 2012)
*Shewanella* species	Water/soil exposure	Cr(VI) reduction capacity, oxidative stress response, and DNA repair gene induction	Enzymatic reduction of Cr(VI) to Cr(III), ROS activation, detox enzymes, and efflux pumps	(Viti et al., 2014)
*Arthrobacter* species	Soil exposure	Resistance to Cr(VI), DNA repair, metabolic adaptation	Chromate efflux (chrA gene), enzymatic reduction, and antioxidant defense.	(Viti et al., 2014)
Algae (*Scenedesmus, Chlorella*)	Aqueous environment	Growth inhibition, photosynthesis impairment	ROS generation, inhibition of the electron transport chains	(Cervantes et al., 2001)
Mixed rhizospheric bacteria	Soil/plant root association	Reduction in plant growth-promoting activities without reduced bacterial growth	Metabolic disruption, gene expression alteration affecting IAA, and siderophore production	(Kumar et al., 2020; Rahman and Thomas, 2021)

## 3 Current approaches for Cr(VI) remediation in water bodies

Cr(VI) is regarded as a carcinogen with several toxicological implications on the living organisms. Moreover, its high solubility and mobility in the aqueous medium aggravate the toxicity to a much greater extent, requiring advanced remediation approaches. As a result, the remediation of Cr(VI) contaminated water bodies has become a critical environmental priority, with physical, chemical, microbial, and plant-based approaches being the most commonly explored strategies. Physical remediation includes Cr(VI) adsorption onto less harmful or non-toxic adsorbents. The efficiency of Cr(VI) adsorption ranges between 80 – 99%, with faster adsorption rates being quite evident with increased Cr concentration (Yan et al., 2020). The major demerits of the process lie in the cost involved in the use and reuse of adsorbents, as well as the recovery of Cr thereafter. The process is also labor and resource-intensive. Several research studies have been carried out on this aspect and are documented under **Supplementary Table 4**.

Chemical remediation focuses on redox reactions that reduce toxic Cr(VI) to its less harmful trivalent form, Cr(III). This approach is generally rapid, cost-effective, and largely independent of the initial chromium concentration, making it attractive for controlled treatment systems (Ma et al., 2024b). However, chemical methods often generate secondary pollutants, such as chemical sludge or residual reagents which can introduce new environmental concerns and limit their suitability for large-scale or long-term applications.

Microbial remediation offers a biologically driven alternative by harnessing the natural ability of certain bacteria to interact with Cr(VI) through biosorption, bioaccumulation, and enzymatic biotransformation (Jobby et al., 2018; Sharma et al., 2022). Biosorption is a passive, energy-independent process in which microbial cells bind Cr(VI) ions on their surface, thereby reducing metal mobility and bioavailability. Both living and dead cells can participate in this process, although dead cells are often more effective. A notable limitation of biosorption is its reduced selectivity in the presence of competing metals, such as cadmium, which can compromise overall efficiency.

In contrast, bioaccumulation is an active, energy-dependent process involving the transport of Cr(VI) ions into living microbial cells. This uptake often occurs through sulfate transport pathways, as Cr(VI) structurally resembles sulfate ions (Thatoi et al., 2014). Once inside the cell, Cr(VI) can undergo biotransformation, where enzymatic systems reduce it to Cr(III). This detoxification process is regulated by specific genes, such as *chrR*, which mediates intracellular reduction, and *chrA*, which facilitates the efflux of reduced chromium from the cell (Arishi and Mashhour, 2021). Although microbial remediation is environmentally friendly, energy-efficient, and cost-effective, its practical application is limited in large or dynamic water bodies. Furthermore, introducing specific microbial strains may disrupt native microbial communities and alter ecosystem balance.

In recent years, bamboo-based remediation has gained attention as a sustainable and scalable alternative that bridges biological and physicochemical approaches. Bamboo plants are capable of removing Cr(VI) *in situ* through phytoremediation, simultaneously improving ecosystem health while extracting contaminants from soil and water (Liang et al., 2022). In addition, bamboo-derived materials such as biochar and activated carbon exhibit high adsorption capacities at relatively low cost and with minimal environmental impact (Das and Sarma, 2025; Kumar et al., 2025a). Owing to its rapid growth rate, high biomass yield, extensive root system, and adaptability to diverse environmental conditions, bamboo offers a versatile remediation platform that overcomes many of the technical and ecological limitations associated with conventional methods.

A comparative evaluation of these remediation strategies, presented in **Table 9**, highlights their mechanisms, advantages, limitations, and scalability. While physical adsorption techniques achieve high removal efficiencies, challenges related to adsorbent regeneration and chromium recovery remain significant barriers (Assefa et al., 2024; Haroon et al., 2025). Chemical reduction methods are effective and economical at small scales but often compromise environmental sustainability due to secondary pollution (Ma et al., 2024b). Microbial approaches, though eco-friendly and efficient under controlled conditions, face stability and scalability issues in natural water systems (Pradhan et al., 2017; Ma et al., 2024b). In contrast, bamboo-based remediation strategies demonstrate superior environmental compatibility, scalability, and long-term applicability, making them a highly promising solution for sustainable Cr(VI) remediation. With its rapid growth, high biomass production, extensive root network, and adaptability to diverse environments, bamboo provides a multifaceted remediation platform that addresses many of the technical and ecological shortcomings of conventional Cr(VI) removal methods.

**Table 9:** Comparative assessment of Cr(VI) remediation strategies.

Remediation strategy	Primary mechanism	Key advantages	Major limitations	Scalability	Environmental impact	Ref.
Physical adsorption (activated carbon, biochar, nanoadsorbents)	Surface adsorption via electrostatic attraction, ion exchange	High removal efficiency, rapid treatment, simple operation	High cost of adsorbent synthesis, regeneration difficulty, post treatment disposal issues, reduced efficiency in multi-ion system	Moderate	Secondary waste generation, energy-intensive preparation	(Mi and Wang, 2024; Haroon et al., 2025)
Chemical remediation (reduction-precipitation, coagulation, electrochemical)	Chemical reduction of Cr(VI) to Cr(III) followed by precipitation	Fast reaction rates, effective in controlled systems	Sludge formation, reagent consumption, additional treatment needed	Moderate to high	Potential chemical residue accumulation	(Jin et al., 2016; Verma and Balomajumder, 2020)
Microbial remediation (bacteria, fungi, algae)	Enzymatic reduction of Cr(VI) to Cr(III)	Ecofriendly, low energy demand, selective reduction	Sensitive to pH, temperature, toxicity, difficult to maintain activity at large scale	Low	Environmentally benign	(Pattnaik et al., 2022; Dubey et al., 2024)
Phytoremediation (terrestrial and aquatic plants)	Uptake accumulation, and reduction within plant tissues	*Insitu* treatment, ecosystem restoration, cost effective	Slow remediation rate, biomass handling, limited metal tolerance	Moderate	Highly sustainable	(Sharma et al., 2023a; Basu et al., 2024)
Bamboo based remediation (live bamboo, bamboo biochar, activated carbon)	Combined plant uptake, rhizofiltration, and adsorption	Rapid growth, high biomass, dual remediation pathways, long term stability	Requires land area, biomass management	High	Low environmental footprint, carbon sequestration	(Were et al., 2017; Bian et al., 2023; Rani et al., 2023b)

## 4 Bamboo – distribution, characteristic properties, environmental benefits/applications, invasiveness and associated problems

Bamboo is widely distributed in India and globally, with distinct regional distributions influenced by climate and geography. While bamboo is present in nearly all Indian states, its distribution and density differ primarily due to variations in climate and soil conditions. The north eastern states of India (Arunachal Pradesh, Assam, Manipur, Meghalaya, Mizoram, Nagaland, Sikkim, Tripura, and West Bengal) are the richest in Bamboo species diversity, hosting more than 50% of India’s bamboo species (Das et al., 2023). Other major regions are the Western and Eastern Ghats, the Andaman and Nicobar Islands, and some central and southern parts like Madhya Pradesh, Uttarakhand, Andhra Pradesh, Kerala, Karnataka, Maharashtra, and Tamil Nadu. India has around 136–144 species of bamboo across multiple genera, growing in tropical, subtropical, and temperate regions up to altitudes of 4000 meters (Dutta et al., 2025). The subject area of bamboo is 9.57 million hectares of the Indian forests, which constitute nearly 12.8% of the overall forest cover in the country; a comparatively large source of bamboo in the world (Kumar et al., 2023c).

The bamboo species are scattered on the five continents but the main areas are tropical, subtropical, and temperate countries. There are about 1678 species in 123 genera (Ahmad et al., 2021b; Williams et al., 2024). The Asia-Pacific, the America, and the Africa are the main bamboo bearing areas. In the Asia-Pacific, China, India, Myanmar, Thailand and Japan are the greatest contributors. Bamboo is concentrated in Asia which has 65% of the world resources and India and China are the global leaders in terms of area and species diversity. Bamboo is found in the southeastern United States to Central America and South America, extending to Brazil, Colombia, Venezuela, and Mexico, although the latter is very diverse (particularly in the Amazon basin). Mozambique, Sudan, Madagascar, Senegal, Nigeria, and the Congo Basin are home to bamboo in Africa, with Madagascar the most biodiverse place (Lee et al., 2023; Bamboos of the World, 2025).

Bamboo thrives in moist, warm climates but can also grow in mountain and temperate regions. Some bamboo species exist at high elevations up to 4000 meters (Dutta et al., 2025). The total area of the bamboo forests consists of over 36 million hectares throughout the world, the majority of which are located in Asia, especially in the countries of India and China, then in Latin America and Africa (Worldwide distribution of bamboo, 2025). Distinctive characteristic features of bamboo make it versatile and valuable for various applications. Physical properties of Bamboo vary according to species and stem position. Its density ranges from about 0.54 to 0.76g/cm^3^, typically increasing along the length of the stem due to the higher concentration of vascular bundles in the upper sections. Moisture content decreases from bottom to top in the stem (Hartono et al., 2022).

Extraordinary mechanical properties of bamboo include compressive strength ranging from 35 to 499 kg/cm² and tensile strength between 932 and 2868 kg/cm² (Suriani, 2020). Shear strength varies from 32 to 77 kg/cm^2^ and is influenced by moisture content and structural composition. The overall mechanical strength of bamboo increases from the base to the apex of the stem, primarily due to the distribution of vascular bundles, which also affects its density (Hartono et al., 2022). Bamboo fibers have less lignin and hemicellulose compared to wood, and are mostly made of cellulose (Kumar et al., 2023b). They are naturally decomposable, antibacterial, UV protective, soft, breathable, eco-friendly, lightweight, and have a high tensile strength. Outer layer of bamboo have higher cellulose content and fiber quality varies according to the species and plant part (Irawan et al., 2025).

The uses of bamboo are very diverse and it provides significant environmental impacts that sustain resilience and stability of the ecosystem. It is also significantly more helpful in attenuating climate change than most tree species due to its high level of carbon dioxide capture, up to 2 tons over a period of seven years and up to 12 tons per hectare yearly. Meanwhile, bamboo produces 35% more oxygen than a tree, thus improving air quality significantly. The deep root and rhizome network stabilizes the soil and prevents erosion which protects slopes, riverbanks and land slopes susceptible to erosion. Further, the speed of the growth and the generational ability of bamboo allows the biomass production to be sustainable with resources not being depleted (Emamverdian et al., 2020). Furthermore, Also, bamboo enables the recycling of the polluted soil and water via phytoremediation, taking up the heavy metals and other industrial contaminants, and consequently improving the environment in terms of health and sustainability.

Bamboo is a resource that can be used in the term of sustainability because it has a variety of applications concerning its environmental usage. It has a smaller carbon footprint compared to the traditional timber or concrete; and is often used with other building materials that are environmentally friendly. Bamboo is a renewable bioenergy source that can be used to make biofuel, charcoal, and pellets, which helps to meet clean energy targets and lessen reliance on fossil fuels. Moreover, bamboo can be employed for land restoration especially the marginal or steep or diminished soils where other crops have difficulties in growing; this helps in improving the biodiversity and recovery of the ecosystem (Patel et al., 2025). Bamboo also contributes to sustainable rural development, as it gives people economic opportunities and subsistence in rural and developing areas. Its fibers are utilized in paper, packaging, and textiles, offering biodegradable substitutes that lessen the pollution caused by plastic.

Bamboo can be invasive, especially certain species with running rhizomes like Phyllostachys, which spread rapidly and form dense monocultures. This high degree of invasiveness may overpower native vegetation by competing with native plant species in the amount of sunlight, water, and nutrients, negatively affecting the biodiversity and causing changes in the ecosystem functions (Chen et al., 2022). Bamboo can displace indigenous plants and possibly destroy habitats due to its quick vegetative reproduction, which makes it even more invasive. The local ecosystem may be further impacted by invasiveness to alter soil hydrology and composition (Canavan et al., 2017).

Although total biomass may not always decline significantly due to increased bamboo stem density. The intensification of Moso bamboo invasion in China has been linked to simplification of forest community structure, decline in species richness, and species dominance shifts. According to (Ouyang et al., 2025), bamboo invasiveness can have a serious negative influence on habitat quality and plant variety. Management strategies for invasive bamboo include selecting non-invasive cultivars, utilizing root barriers, and eliminating uncontrolled growth in order to prevent ecological and financial consequences. The invasive potential is also linked more to human usage and introduction patterns than inherent species traits, with some genera like Bambusa and Phyllostachys being more commonly invasive outside their native ranges (Xu et al., 2020). While bamboo offers many environmental benefits, its invasiveness poses ecological challenges that require careful monitoring and management.

## 5 Bamboo as a phytoremedial tool for the remediation of Cr(VI) contaminated environment

Bamboo has emerged as a promising phytoremediation tool for environments contaminated with hexavalent chromium (Cr(VI)) due to its remarkable tolerance to heavy metals and rapid biomass growth. The phytoremediation potential of bamboo lies mainly in its ability to absorb Cr(VI) from contaminated soil and water through its root system. Once absorbed, bamboo can either stabilize chromium in its roots or translocate it to the above-ground parts based on the species and surrounding environmental factors (Ranieri et al., 2021). This mechanism helps reduce the bioavailability and mobility of the toxic Cr(VI) form in the environment. Bamboo’s roots play a crucial role in the initial chromium uptake, where Cr(VI) is often enzymatically reduced to trivalent chromium (Cr(III)), a less toxic and less soluble form (Were et al., 2017). This reduction process significantly lowers chromium toxicity and environmental risks by immobilizing chromium in soil and preventing its leaching into groundwater or uptake by other organisms.

The ability of bamboo to grow in diverse soil types and harsh conditions further supports its application in chromium-contaminated sites. Bamboo’s extensive and dense root network not only aids in chromium absorption but also stabilizes the soil, preventing erosion and the spread of contaminants through dust and runoff. *Bambusa vulgaris* and *Dendrocalamus asper*, have been shown to accumulate considerable amounts of chromium primarily in their roots, limiting translocation to aerial parts (Bian et al., 2020). This feature is beneficial for phytostabilization, where contaminants are immobilized within the root zone, reducing environmental dispersal and exposure risks. However, some species can translocate chromium to shoots, which opens up possibilities for phytoextraction—removing contaminants from soil by harvesting the above-ground biomass. Phytoextraction provides a practical way to clean contaminated sites over successive planting and harvesting cycles.

In addition to chromium removal, bamboo’s fast growth and high biomass production make it a sustainable option for phytoremediation projects (Sharma et al., 2023a). The harvested biomass containing accumulated chromium can be managed carefully to avoid secondary pollution, such as by incineration or safe disposal (Were et al., 2017). Moreover, bamboo plantations on contaminated soils can rehabilitate degraded land, improve soil texture and organic content, and create green barriers that mitigate pollution spread. Overall, bamboo represents an economical, eco-friendly, and efficient strategy for remediating Cr(VI)-polluted environments by combining phytostabilization and phytoextraction mechanisms, making it a valuable plant resource in environmental management and pollution control efforts (Ranieri et al., 2021). The **Table 10** provides a comparative account of bamboo with other commonly used hyperaccumulator plants thus highlighting its advantages.

**Table 10:** Comparison of phytoremedial potential of bamboo with other plant hyperaccumulators.

Plant	Growth rate	Root system	Growing season	Biomass	Ref.
Bamboo	Very fast	Very deep and dense root network	Perennial	Very high root and shoot biomass	(Emamverdian et al., 2020)
*Brasicca juncea*	Reduced yield, stunted growth	Roots penetrate shallow depth, Unable to work in deeply polluted soils	Perennial, Requires multiple growing seasons	Low biomass in roots and shoots	(Bortoloti and Baron, 2022)
*Leersia hexandra*	Slow growth rate	Roots penetrate shallow depth, Unable to work in deeply polluted soils	Perennial, Multiple growing seasons required	Modest dry matter	(Liu et al., 2011; Lin et al., 2019)
*Pongamia pinnata*	Slow growth, Slower maturation	Root affected by metal contaminants	Perennial	Stunted biomass growth	(Das et al., 2022)
*Convolvulus arvensis*	Grows aggressively as a weed	Roots penetrate shallow depths, Not suitable for deeper soil contamination	Perennial	Low above ground mass	(Wani et al., 2023)

The mechanism of chromium (Cr(VI)) remediation by bamboo involves several key steps that enable this plant to tolerate, stabilize, and extract chromium from contaminated environments. When bamboo is planted in Cr(VI)-contaminated soil, the chromium ions are absorbed primarily by the roots. The roots serve as the primary site for chromium uptake where an important reduction reaction takes place, leading to the conversion of Cr(VI) into the less toxic (Cr(III)). This enzymatic or chemical reduction within the root zone lowers the toxicity of chromium and prevents its easy movement through the soil or into the plant’s aerial parts (Sharma et al., 2023a).

Bamboo species exhibit different strategies after chromium uptake. Several species such as *Dendrocalamus asper*, *Bambusa vulgaris*, and *Dendrocalamus membranaceus* limit the translocation of chromium from roots to shoots, which effectively stabilizes the metal within the root zone and soil around the roots—a process known as phytostabilization. This stabilization reduces the bioavailability and environmental mobility of chromium, thereby preventing it from entering the groundwater or food chain. Meanwhile, certain species like Bambusa bambos have demonstrated potential for phytoextraction, where chromium is translocated to aerial parts, enabling removal of the contaminant by harvesting the above-ground biomass (Zhang et al., 2021).

Bamboo’s extensive root system also contributes to environmental remediation by preventing soil erosion and reducing the spread of chromium contaminants through surface runoff or wind erosion. The dense clumping growth habit of bamboo further aids this soil stabilization function. Furthermore, soil pH and microbial communities in the bamboo rhizosphere can influence chromium mobility and the effectiveness of phytoremediation. An increase in soil pH tends to decrease chromium solubility and bioavailability, enhancing plant tolerance and remediation efficiency (Were et al., 2017).

**Figure 3:**
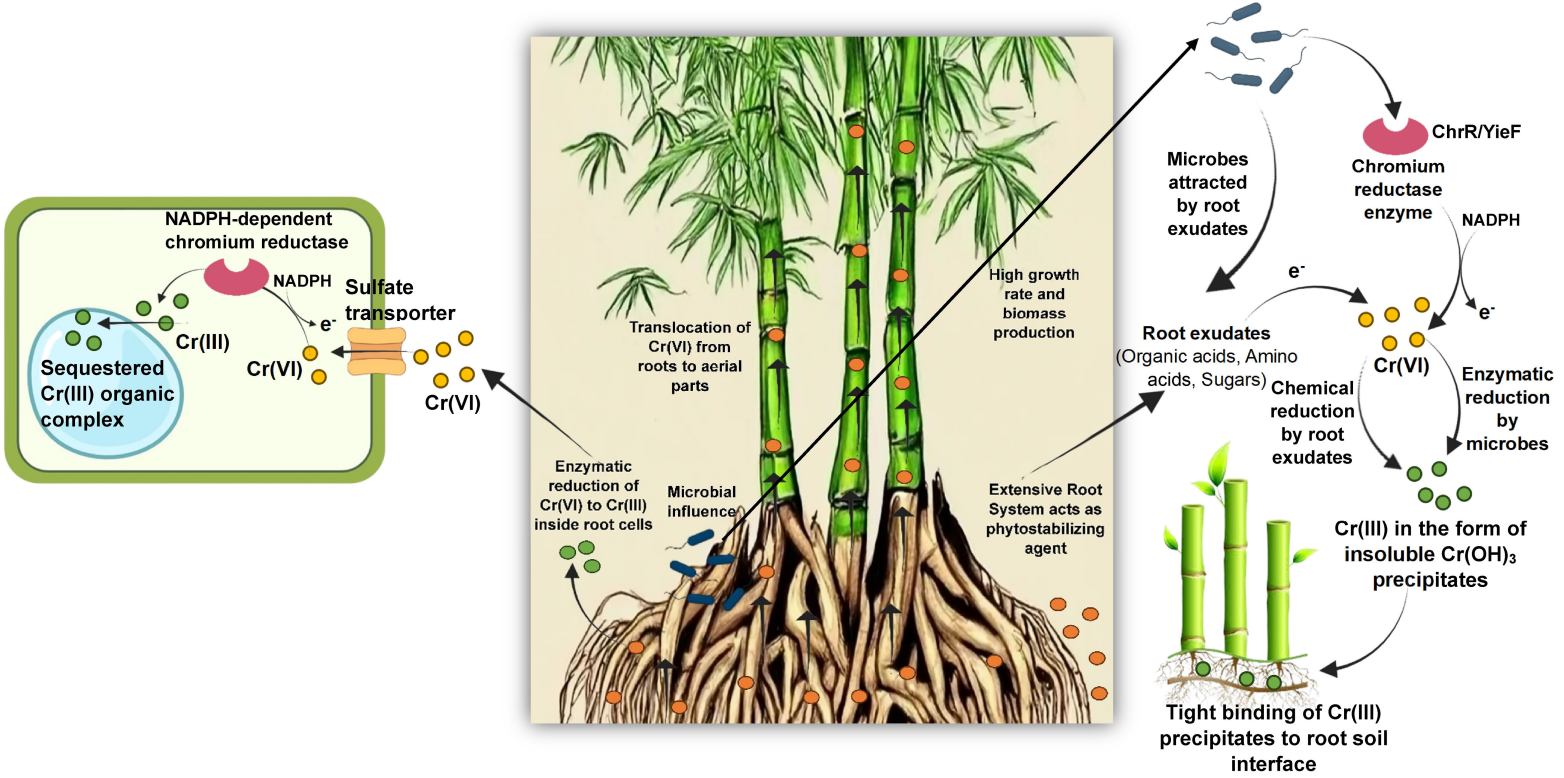
Phytoremediation mechanisms of Cr(VI) in the bamboo plant.

In summary, bamboo remediates Cr(VI) contamination by absorbing chromium through roots, reducing toxic Cr(VI) to Cr(III), stabilizing chromium primarily in the root zone (phytostabilization), and in some cases, translocating chromium to shoots for phytoextraction (**Figure 3**). Its robust growth, high biomass, and root architecture make it an environmentally friendly and effective phytoremediation tool for chromium-contaminated sites.

### 5.1 Bamboo as a natural adsorbent of chromium

Chromium (Cr) occupies the seventh position in terms of abundance on earth and is the first carcinogenic element and the fifth potentially toxic element (Kumar et al., 2020). The high concentrations of Cr in the surrounding environment have recently drawn public attention to environmental pollution. Economical, eco-friendly, and useful remediation technologies for Cr(VI) have been developed by numerous researchers in recent years. Many researchers from different countries have used different species of bamboo to phytoremediate soil contaminated with heavy metals. The genera *Phyllostachys*, *Pleioblastus*, *Dendrocalamus*, *Indocalamus*, *Sasa*, *Gigantocloa*, and *Bambusa* are frequently employed for phytoremediation purposes. With significant biomass production contamination, they demonstrated a high capacity for the heavy metal accumulation like Cd, Pb, Zn, Cu, Si, Cr, and Fe; biomaterial remediation is regarded as a viable and alternative method in this regard (Yan et al., 2020).

Bamboo is a widely available resource, rapidly growing, renewable, and beneficial to the environment. Bamboo has been used for ecological restoration in a number of places around the world, including Africa, where it has been effectively used for wastewater treatment at Murchison Bay prison in Luzira, land rehabilitation, and the treatment of polluted Lake Victoria (Kirunda, 2005). The ecological attributes of a critically degraded basaltic site in Jabalpur, India, are improved by agroforestry models based on bamboo (Behari et al., 2000). *Bambusa bamboos*, *B. nutans*, and *Dendrocalamus strictus* were successfully incorporated into seven agroforestry practices aimed at reversing land degradation and restoring of degraded agricultural lands in central India (Singh et al., 2020).

*Phyllostachys pubescens* (Bambusa) exhibits shown a remarkable ability to grow in 180 mg Cr/L at a 600 mm/year irrigation flow rate. Its capacity for phytostabilization was demonstrated by the fact that, following six weeks of cultivation, the majority of the Cr bioaccumulation was found in the below-ground sections. Bamboo’s aerial portions exhibited increased Cr bioaccumulation and a high translocation factor following 12 weeks of cultivation (Ranieri et al., 2023). According to reports, *P. pubescens* removed about 42% and 60.7% Cr from the soil at 6 and 12 weeks, respectively, from a starting soil concentration of about 300 mg/kg Cr to a final soil concentration of about 118 mg Cr/kg (Ranieri et al., 2021). This suggests *P. pubescens* works by using a blend of two mechanisms, phytostabilization and high extraction potential. In the initial period, it acts as a phytostabilizer, thereby sequestering Cr in its roots, followed by a shift towards the phytoextraction mechanism later. According to Ranieri et al., *P. pubescens* cultivated at a soil having about 100 mg Cr/kg, had a 100% survival rate. This suggests a higher tolerance of the species towards Cr. Furthermore, they discovered that the bamboo’s roots had higher concentrations of Cr(1.31 mg/g d.wt.) than its aerial parts (0.86 mg/g d.wt.), suggesting the possibility of phytostabilization and phytodegradation. This backs the use of *P. pubescens* may be able to tolerate higher levels of metal stress and be used as a phytoremediation agent for soil contaminated with Cr at concentrations as high as 200–300 mg/kg. Other bamboo species like *Bambusa vulgaris*, *B. blumeana*, *B. bambos*, *Dendrocalamus asper*, *D. birmanicus*, and *D. membranaceus* exhibited a 100% survival rate when planted in chromium-contaminated tannery soils in Kenya, except *D. birmanicus*, which had an 83.3% survival rate. This signifies that bamboo species remediation capabilities are not limited to specific soil types or geographical regions. The translocation factor of B. bambos was more than 1, suggesting the possibility of phytoextraction with accumulation between 1337 and 3398 mg/kg Cr (Were et al., 2017). According to (Sinha et al., 2020), the concentration in the above-ground parts varied from 1.87 to 34.44 mg/kg d.wt. This confirms the high Cr extraction potential of *B. bambos* and *D. strictus*, thereby suggesting that the mechanism may vary from one bamboo species to the other. It can be concluded that species like *P. pubescens* are an ideal tool for the cleanup of Cr from the soil via phytostabilization and degradation, while certain species like *B. bambos* and *D. strictus* can act as efficient hyperaccumulators for rapid extraction of Cr.

### 5.2 Bamboo-based adsorbents and their preparation

The use of bamboo-based adsorbents such as bamboo biochar, bamboo charcoal, activated carbon and modified bamboo etc., is gaining increasing recognition owing their potentiality in several adsorptive processes. The bamboo-based adsorbents, are produced from the rapidly growing bamboo plant which is characterized by high surface areas, porous structure and abundance of functional groups that helps them to adsorb the impurities. Bamboo based adsorbents are applied in the removal of natural impurities, dyes, and toxic metals from water. The bamboo-based adsorbents are designed to be potential utilities for solving various environmental problems due to their eco-friendly nature and justifiable utilities in air pollution and water purification (Alfei and Pandoli, 2024). Bamboo is an eco-friendly and renewable source which lowers the ecological impact of procedures for water treatment and lessen the need for prepared adsorbents. The availability and cheapness of bamboo make it a viable choice for widespread applications, especially in countries where a particular crisis of contamination of water exists. Bamboo based adsorbents are successful in their functioning, due to the various functional groups which improve their binding ability such as carboxyl, hydroxyl and phenolic functional groups. In addition, the ability of bamboo as an adsorbent and selection for specific pollutants can be further increased by chemical and physical modification. These substances are produced from bamboo biomass using methods that increase their surface area and porous structure, such as pyrolysis, carbonization, and torrefaction (Lou et al., 2023). Pyrolysis is the main process used to produce biochar, charcoal, and activated carbon; however, the conditions and subsequent treatments vary greatly. The process of slow pyrolysis, which produces biochar, consists of subjecting the biomass to moderate heating (300 - 700°C) under oxygen-free conditions. Also, a carbonization process, but at a somewhat higher temperature between 400 and 800°C, is used to produce charcoal (Phounglamcheik et al., 2020). Mostly, activated carbon is produced by pyrolyzing followed by activating the biomass or materials rich in carbon. The activation takes place in the presence of activating solutions at a temperature exceeding 600°C (Wang et al., 2020b; Erdem and Dogru, 2021). This activation favors the augmentation of the surface area of the structure and produces a very porous material.

#### 5.2.1 Biochar

Biochar (BC) is defined as “the solid material obtained from the thermochemical conversion of biomass in an oxygen-limited environment” (Antanasković et al., 2025) by the International Biochar Initiative (IBI). Practically speaking, BC is the light, black residue that is principally consisting of carbon and ash, while also being rich in aromatic structures and mineral content. It is made when organic material biomass-such as wood, manure, leaves, garbage, etc. are decomposed thermally in the presence or absence of oxygen (O_2_). Pyrolysis is the term employed to describe such a thermal decomposition process. The most widely employed pyrolysis temperature is in the 400–600°C temperature zone (Panwar et al., 2019).

Biochar can serve as a valuable sorbent application in the area of water and wastewater treatment owing to its ability to effectively eliminate several environmental contaminations like heavy metals, nutrients, and organic pollutants (Barquilha and Braga, 2021; Gopinath et al., 2021; Zhang et al., 2023). It is a viable, carbon-rich and stable product formed from thermal processing of all sorts of biomass or carbonaceous materials (Singh et al., 2021). Biochar can be produced from various feed stocks like rice straw, rice husk (Rubeena et al., 2018), black gram, Lantana camara & maize stalk (Das et al., 2023), crop residues (Anand et al., 2022), micro algal biomass (Nakarmi et al., 2022), etc. Biochar can be produced from a variety of thermochemical conversions like torrefaction, flash carbonization, hydrothermal carbonization, gasification, and pyrolysis (Nidheesh et al., 2021; Kumar et al., 2023a). Bamboo biochar is a popular sorbent based on bamboo used for removal of heavy metals from water. Its higher effectiveness of Cr removal has been reported by Singh et al. (2021) with increase in pyrolysis temperature (Singh et al., 2021). Bamboo biochar was produced from bamboo saw dust by pyrolysis. Magnetic bamboo biochar (MBB) was made using this bamboo charcoal by co-precipitation method. The Chitosan modified magnetic bamboo biochar (CMBB) was made out of it by forming glutaraldehyde cross link. The absorption capacity of the bamboo biochar was increased when it was converted to the magnetic bamboo biochar by co-precipitation method (Zhang et al., 2020a). The highest adsorption capacities of magnetic bamboo biochar (MBB) and chitosan-modified magnetic bamboo biochar (CMBB) for Cr(VI) were determined to be 75.8 mg/g and 127 mg/g, respectively, at 25°C (Zhang et al., 2020a). It was found that CMBB was more effective than MBB in the removal of Cr(VI) because it possessed a higher capacity for the removal of Cr(VI) (127 mg/g) also over a wider range of pH (2-10). Also, the high regeneration of CMBB makes it an effective adsorbent in the detoxification of Cr(VI) from waste waters.

According to the findings of Lo et al. (2012), the activated carbon created from Moso and Ma bamboo had an effectiveness of almost 100% and 91.7% for the removal of the heavy metals Cu^2+^, Cd^2+^ and Cr^3+^, respectively, after one and two activations by adsorption (Lo et al., 2012). The absorbents themselves and the use of vermiculite were established to be most effective in the removal of the hexavalent chromium from a sample of effluent, which had a chromium content of 184.8 mg/L, after having been subjected to treatment through columns, containing various absorbent materials. The combination of vermiculite and coir pith reduced the amount of total chromium, to 184.8-4.48 mg/L considerably (Tripathi and Chaurasia, 2020). The alkaline KOH activated carbon derived from bamboo (*Oxytenanthera abyssinica*) waste displayed an adsorption efficiency of 98.28% and a capacity of 59.23 mg/g, at pH-2, when investigated for its capability to remove Cr(VI) from aqueous solution. In addition to its excellent adsorptive properties, the magnetic biochar has other advantages as compared with other forms of biochar or biochar composites, in virtue of its easy recoverability from solution by means of magnetic separation.

Modified ethylenediaminetetraacetic acid (EDTA) intercalated Mg/Al layered double hydroxides (LDH) biochar gave excellent Cr(VI) removal. Modified EDTA-LDH biochar was prepared by liquid phase coprecipitation of LDH on biochar substrates. The existing forms of chromium (Cr) in the environment are mainly in hexavalent (Cr(VI)) and less harmful trivalent (Cr(III)) oxidation states. Bamboo char supported Zero-valent iron (BB/ZVI) was used (Zhou et al., 2020) for the reduction of Cr(VI). An effective detoxification procedure for Cr(VI) in affected environments is the combination of adsorption and reduction. Zero-valent iron (ZVI) was found to be the ideal sorbent for the removal of Cr(VI) by means of adsorption and reduction. BB/ZVI was found to reduce the Cr(VI) by 1.7 times compared to normal bamboo biochar. Thus, BB/ZVI had greater electron transfer than bamboo biochar (2.5 μA e-) compared to normal bamboo biochar (0.5 μA e-).

The mechanism of adsorption is presented by four different pathways: (i) solute moving from the bulk of the solution to the surface of the adsorbent (bulk diffusion); (ii) solute externally mass transferred across the liquid film to the exterior surface of the adsorbent (film diffusion); (iii) solute moving from the exterior surface into the porous adsorbent (intraparticle diffusion or pore diffusion); (iv) solute being adsorbed on active sites of the interior and exterior of the adsorbent (Ma et al., 2012). The first stage cannot be regarded as a rate controlling step, since it is generally considered to be a fast process. It may be considered that the pore diffusions and film diffusions are accentuated as rate controlling steps, which are influenced by temperature, so that the sorption process is favored at higher temperatures as demonstrated by increased capacities and rates. In a variety of adsorption systems pseudo-second order kinetics are recommended by the literature review (Tewari et al., 2018).

#### 5.2.2 Hydrochar

Wet pyrolysis, also referred to as hydrothermal carbonization, produces hydrochar, a solid byproduct (Allohverdi et al., 2021). Hydrothermal processes like hydrothermal liquefaction (HTL) and hydrothermal carbonization (HTC) are used to create hydrochar, a unique type of biochar. Because of their beneficial pore volume, surface area, regeneration capacity, and high efficiency, hydrochar are a good substitute for cleaning up a range of pollutants (Ighalo et al., 2022). HTC usually occurs between 180 and 240°C for 5 to 240 minutes at subcritical water pressures. Due to its lower carbon conversion, hydrochar has larger atomic ratios of hydrogen-to-carbon and oxygen-to-carbon than biochar (Masoumi et al., 2021).

The usual product distribution from hydrothermal carbonization is 40–70% hydrochar, 10–20% liquid, and 2–5% gases, depending on the dry basis of the input feedstock. HTL is performed at higher temperatures, typically between 250 and 375°C, and pressures that range between 10 and 22 MPa. Biocrude oil is the primary product produced during the HTL, with smaller amounts of syngas and hydrochar (Masoumi et al., 2021). Polyethyleneimine-modified hydrochar (PEI-HC) was produced by hydrothermally carbonizing (HTC) bamboo and methyl acrylate with ammonium persulfate acting as an initiator.

The resulting hydrochar was then treated with polyethyleneimine (PEI) and used to treat Cr(VI). Characterization revealed that PEI was effectively grafted onto the hydrochar, and the abundant nitrogen and oxygen functional groups in PEI-HC enhanced its Cr(VI) adsorption capacity (Li et al., 2021). Two modified hydrochar were created by co-hydrothermally carbonizing bamboo sawdust with either zinc chloride (ZnCl_2_) or aluminium chloride (AlCl_3_) at 200°C for seven hours. With increased surface area, pore structure, and aromaticity, the modified hydrochar performed better than the unmodified hydrochar in the extraction of Cr(VI) from aqueous solutions (Li et al., 2021).

#### 5.2.3 Activated carbon

Activated carbon (AC) is a carbonaceous highly porous adsorbent solid having a high surface area, black color, and no taste (Mondal and Garg, 2017; Kumar et al., 2023a; Rathi et al., 2024). Various starting materials, such as rubber wood saw dust, ramie fiber and areca husk (Nath et al., 2023), shells of almond (Singh et al., 2023), peanut skin (Mandal et al., 2024), agricultural waste (Chauhan et al., 2023), and Calotropis gigantea stem (Sahu et al., 2023) are available for the preparation of activated carbon. The starting materials for the preparation of activated carbon should be highly carbonaceous. Activated carbon is used in water and wastewater treatment (Kaur et al., 2024), air filters (Nam et al., 2018; Maniarasu et al., 2023), and energy applications (Ramalingam et al., 2024).

Prior to their use in activated carbon production, raw materials undergo preprocessing or pretreatment steps such as washing, drying, and particle size reduction. After pretreatment, the materials are subjected to thermal conversion processes. The primary thermal conversion methods include pyrolysis and carbonization. The activation is aimed at improving the characteristics of the carbon produced, particularly its porosity and pore structure. Among the methods of activation are physicochemical, chemical and physical (Jjagwe et al., 2021; Ahmad et al., 2022). Asrat et al., 2021 prepared an activated bamboo carbon from species *Arundinaria alpine* to remove Pb from aqueous solution (Asrat et al., 2021). Alfatah et al., prepared an activated carbon from *Bambusa vulgaris* with a large surface area to remove Hg^2+^ using the two-stage activation process with KOH/NaOH mixture (Alfatah et al., 2021). The chars were mixed with KOH/NaOH at different impregnation ratios of 1:1 (AC-1), 1:2 (AC-2), and 1:3 (AC-3) respectively. Bamboo was carbonized at a temperature of 500°C with nitrogen flow. Char was dried and then pyrolyzed at a temperature of 800°C. The chars were washed with 0.1 M HCl to remove unreacted material. The adsorptive capacity of activated bamboo carbon for Hg^2+^ removal is caused by its unique pore shape and active sites (effectiveness). Li et al. (2021) prepared a zeolite-activated carbon from coal gangue so as to remove Cu^2+^ and Rhodamine-B from aqueous solution. It is believed that the efficiency for the adsorption of heavy metals and other contaminants on the bamboo-based zeolite-activated carbon is based on its abundance in macropores, mesopores, and micropores.

#### 5.2.4 Charcoal

Charcoal is the carbonized product of wood pyrolysis, which is chiefly applied as a fuel or reducing agent in industry. As an energy-dense fuel, it has advantages over raw biomass. This product is used by some industries, including pharmaceuticals, cosmetics, fertilizers, animal food, electrical energy production, water purifying and filters (Kabir et al., 2025). In agriculture, charcoal may be prepared by the pyrolysis process at high temperature (Bai et al., 2020). Bamboo charcoal is produced from wood by a process that involves pre-carbonization, carbonization, calcination, etc (Ma et al., 2024c). Bamboo is a very good raw material for charcoal production because of its excellent adsorption properties, large surface area and highly porous nature. The rate of adsorption and surface area of bamboo charcoal is four times and ten times respectively higher than that of ordinary charcoal, hence it is recommended for many uses like water purification, anode for dye sensitive solar cells, blood purification, and as an adsorbent (Chaturvedi et al., 2024). Charcoal has been modified by some researchers to produce better adsorption. For example, it has been shown that modified bamboo charcoal removes iron from water by physical activation (Ariyadi et al., 2021). Charcoal post treatment with potassium permanganate and sodium hydroxide, demonstrated an increase in its capability to remove Zn^2+^, Cu^2+^. The enhanced adsorption capacity following modification results from the incorporation of additional functional groups that are useful for the metal ion reactions. These groups are of the carboxyl (C=O), hydroxyl (C-OH) type. The formation of Mn-O bonds which increase the number of active sites for metal binding with a result of better adsorption is also a feature of the modified charcoal (Ma et al., 2024a). Similar to this, acid-base surface modification with HNO_3_ and KOH improved bamboo charcoal’s capacity to sorb Cd (II) and Cu (II) (Wang et al., 2022). Copper-impregnated HCl-activated charcoal effectively removes a variety of heavy metals, such as Cd, Pb, As, Cr, and others (Thotagamuge et al., 2021).

#### 5.2.5 Modified bamboo

After modifying, bamboo is very effectively created and used as an adsorbent to remove heavy metals so that the problem of raw bamboo which has a lower adsorptive capacity, less porosity, and limited surface functionalization can be surpassed. The changes made to bamboo-based adsorbents mainly focus on increasing the surface area, enhancing porosity, changing the functional groups, etc. in order to make them more effective in the removal of heavy metals from water and soil (Liu et al., 2022). The bamboo adsorbents can be modified in several ways: chemically (acid, base, chitosan coating, surface functionalization, etc.), biologically (activation and hydrothermal treatments, for example), physically (surface functionalization, etc.), and physiochemically (Kumar et al., 2025b).

**Figure 4:**
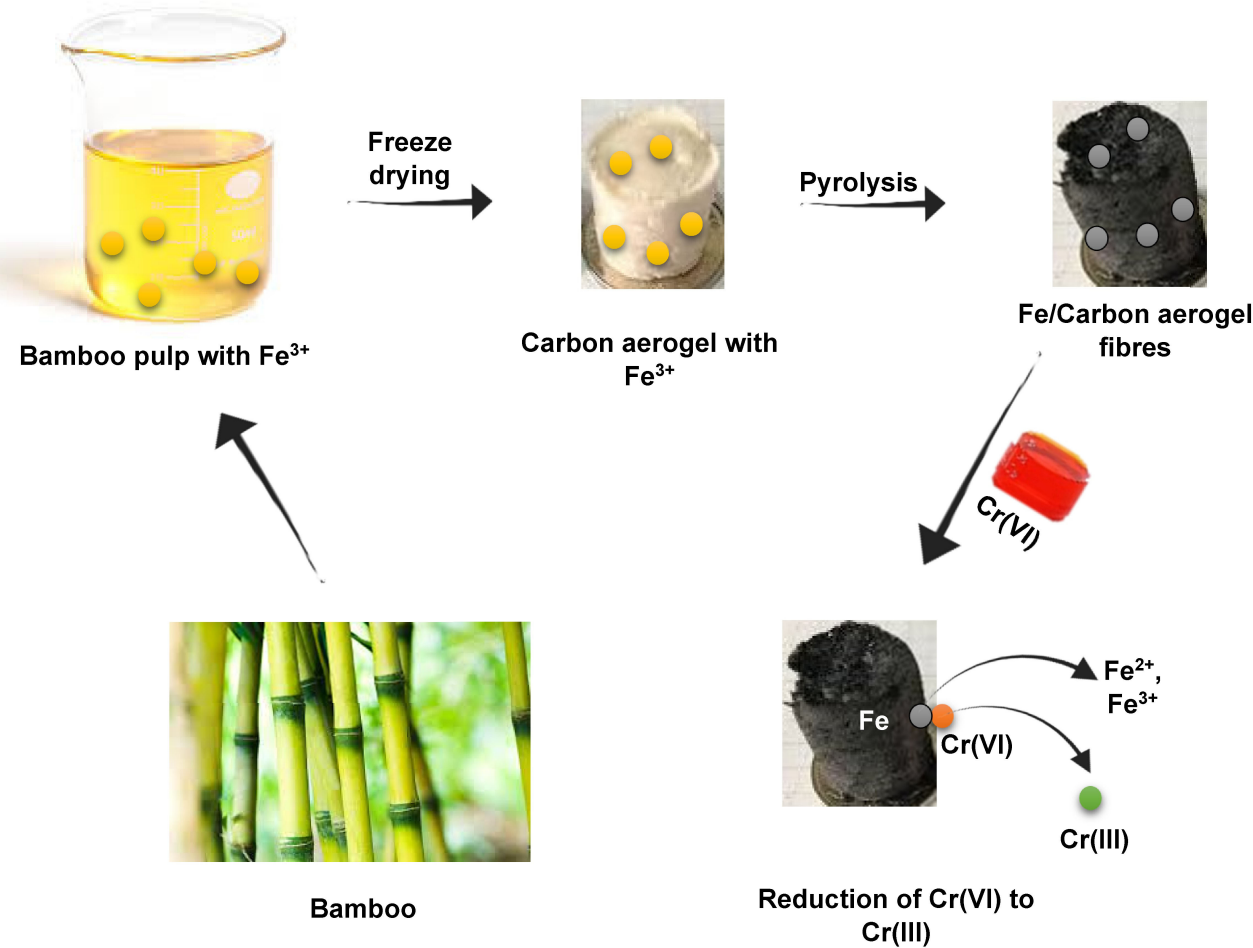
Preparation and working of Fe/carbon aerogel (Fe/CA) for Cr(VI) remediation.

The advantages and disadvantages of the modification process, along with its cost-effectiveness, impact on the environment, and sufficiency, should be thoroughly evaluated before any decision is made. Often, the extraction of heavy metals from water by chemically modifying bamboo is the method of choice. By the addition of Fe(NO_3_)3•9H_2_O, the capacity of bamboo to absorb Cr was significantly enhanced. The bamboo pulp was dispersed in deionized water, combined with Fe(NO_3_)_3_•9H_2_O, freeze-dried, and then pyrolyzed under a controlled condition to obtain the Feo(NO_3_)_3_/cellulose hybrid aerogel (Fe/CA) (**Figure 4**) (Xue et al., 2021).

Quite a large number of Cr(VI) ions that were adsorbed on the surface have been changed into Cr(III), which is a less dangerous nutrient that the body needs for the metabolism of fat and sugar, through the numerous interconnected pores of the adsorbent, thereby resulting in a high adsorption capacity of Cr(VI). Chandrawansha and Arasaretnam, converted bamboo stems into cellulose glycine hydrogels to adsorb metals like Cd^2+^ and Cr^3+^ (Chandrawansha and Arasaretnam, 2024). The hydrogels preferentially absorbed divalent cations over monovalent and trivalent ones. The greatest efficacy of heavy metal adsorption was achieved by the hydrogel that contained 10% glycine.

Dula et al. studied the removal of Cr(VI) from the aqueous media by carbon activated from bamboo (*Oxytenanthera abyssinica*) waste (Dula et al., 2014). Batch experiments were performed as a function of temperature, adsorbent dose, contact time, pH, and initial Cr(VI) concentration. At optimum pH 2.0 and 27°C, removal efficiency and the amount of Cr(VI) adsorbed were 98.3% and 59.2 mg/g, respectively. The kinetic and isotherm models fitted to the pseudo-second-order and the Freundlich models, respectively. The present biosorption thermodynamics state that the removal of Cr(VI) by carbon derived from bamboo waste is exothermic, spontaneous, and feasible at temperatures ranging from 25°C to 45°C.

## 6 Bamboo-derived material vs other commercial adsorbent materials

Essentially, the adsorbent’s many interconnected pores were responsible for the transformations of a large part of the adsorbed Cr(VI) ions into Cr(III), a less hazardous nutrient that the body requires for the metabolism of fat and sugar, thus leading to a high adsorption capacity of Cr(VI). Chandrawansha and Arasaretnam, modified bamboo stems into cellulose glycine hydrogels for the adsorption of metals such as Cd^2+^ and Cr^3+^ (Chandrawansha and Arasaretnam, 2024).The hydrogels preferentially adsorbed divalent cations over monovalent and trivalent ones. The one with 10% glycine was the best hydrogel for heavy metal adsorption.

In the last few years, many different adsorbent materials have evolved to meet the environmental challenge of heavy metal removal from water solutions. These substances are good because of their high surface area to mass ratio which makes the adsorbents very active when a wide range of chemicals are considered mainly heavy metals and other contaminants (Jarawi and Jusoh, 2023). The adsorption capacity of bamboo-based materials can be substantially enhanced through different processes such as carbonization and activation to achieve values that are either slightly lower or comparable to activated carbon (Mistar et al., 2019). It is thus necessary to compare bamboo-based adsorbents with other commonly used absorbents to evaluate their potential fully.

Kharrazi et al. developed a material activated with KOH that removed effectively lead (Pb^3+^) and chromium (Cr^6+^) at a pH of 2.0 (Kharrazi et al., 2020). The adsorption capacities of the material were 113.63 mg/g and 232.56 mg/g, respectively. Carbon activated from Bambusa vulgaris var. Striata exhibited an astonishing adsorption capacity of 218.08 mg/g for Hg^2+^ and might be used as a competitive example for materials derived from bamboo (Mistar et al., 2019). In some cases, very high adsorption capacities have been reported of synthetic nanomaterials like metal-organic frameworks or graphene oxides (Xue et al., 2020), but synthetic nanomaterials are usually less sustainable and more expensive. Chitosan (CS), a naturally formed polymer, is generally taken up because of its adsorption property, but still, it has small mechanical strength, and stability problems, and in addition, it faces difficulties with regeneration due to its small surface area, low porosity, and large mass. Chitosan (CS), a naturally formed polymer, is generally taken up because of its adsorption property. However, it has low mechanical strength, instability, low porosity, difficult regeneration due to its small surface area, and interesting mass transfer resistance (Upadhyay et al., 2021) among other disadvantages. On the flip side, bamboo-based adsorbents represent a viable alternative to materials with similar or even better performance characteristics. The adaptability of bamboo biochar composites, their vast surface area, and their ability to be regenerated make them ideal for not only local but global environmental rehabilitation of different nature. Bamboo is a fast-growing plant that can be grown in various climatic conditions and easily regenerated. Because of the fact that waste bamboo from industries such as construction, paper, and furniture can be recycled, bamboo-based adsorbents are relatively cheap (Pan et al., 2023). Activated carbon and some other traditional adsorbents are generally made from raw materials such as wood or coal. These materials are expensive to handle, which eventually leads to the cost of activated carbon going up as a whole. For example, activated carbon made from coconut shells costs more to produce than its wood-based counterparts (Promdee et al., 2017). Zeolites, together with other adsorbents to be classified, are either produced artificially or by mining natural deposits. However, in both cases, issues of substantial raw material availability and high processing costs arise. Most of the time, carbonization and activation methods utilized in the fabrication of bamboo-based adsorbents require less energy and fewer chemicals than those of traditional adsorbents. For example, bamboo wastes can be transformed into activated carbon through carbonization and steam activation; 800°C for 120 minutes is the best activation temperature (Pan et al., 2023). If mild chemicals are used to chemically activate bamboo, it can certainly lower production costs in comparison with the conventional methods of producing adsorbents that involve chemical impregnation or steam activation. The first thorough economic analysis of the manufacture of activated carbon from bamboo waste has shown that the internal return rates for the independent and integrated production units are 13.0% and 20.1%, correspondingly (León et al., 2020). Separate research has also been undertaken which compared the costs of bioadsorbents and commercial activated carbon. It concluded that although the conventional activated carbon costs INR 500/kg, bioadsorbents could vary their costs from INR 4.4 to INR 36.89/kg. The financial benefit of bioadsorbents, as well as the use of bamboo-based activated carbon in the removal of heavy metals, is revealed by this significant difference in cost (Renu et al., 2017).

## 7 Challenges, future outlook and way forward

Remediation of soil and water contaminated with Cr(VI) using bamboo and bamboo derivatives is no doubt an excellent clean-up strategy. However, the process presents several challenges that must be addressed to make it more sustainable. Most of the bamboo species have a low translocation factor (TF) and bioconcentration factor (BCF) (Ranieri et al., 2021). Therefore, it is quite necessary to select suitable bamboo species that can tolerate and accumulate high Cr(VI) concentrations. Another challenge faced is related to climatic variations. Although the bamboo plant is sturdy, it still cannot grow under all climatic conditions, thus limiting its application as a phytoremediation tool to certain specific tropical and sub-tropical regions. Moreover, the phytoremediation process is quite slow and takes years to reduce the contaminant. This invites further research in the field, with more focus to be put on developing plant variants with high growth and accumulation rates. Understanding the role of microbial rhizospheric interaction with the plant at Cr(VI) contaminated sites will help develop efficient phytoremediation strategies (Zhang et al., 2020a).

Bamboo derivatives like biochar, activated carbon are effective, but they too have to face several problems. Production of biochar or activated carbon from bamboo involves steps like pyrolysis, where high temperatures are involved. This adds up to the energy consumption and costs. The adsorption of negative metal ions like Cr(VI) on bamboo-based bioadsorbents is pH-dependent. Higher adsorption occurs at a low pH, while adsorption capacity decreases at high pH. Therefore, proper care must be taken to maintain the pH throughout the adsorption process. Surface modifications of the bamboo-based bioadsorbents overcome this limitation of specific pH level requirements (Garg et al., 2023) while simultaneously increasing the cost and complexity of the process. Biochar has a characteristic fine size that can clog the columns in a water treatment system, thus limiting its application. Bioadsorbents, once used, must be properly disposed of, thereby ensuring that Cr(VI) adsorbed onto their surfaces do not get back to the environment again (Basnet et al., 2025).

Although laboratory and pilot scale studies strongly support the potential of bamboo and bamboo derived materials for Cr(VI) remediation, their translation to field scale application remains challenging. Practical deployment requires careful optimization and interdisciplinary collaboration. In particular, long term field trials conducted under diverse environmental settings are essential to assess bamboo growth behavior, chromium uptake efficiency, and broader ecological impacts under realistic contamination scenarios (Bian et al., 2020; Premlata, 2025).

The study showcases a true field-scale application through a real-world case study conducted at chromium-contaminated tannery sites in Kenya, where the remediation strategy was implemented directly under natural environmental conditions rather than controlled laboratory settings. The field results showed a substantial reduction in soil chromium levels along with improvements in overall environmental quality, highlighting the practical effectiveness and scalability of the approach. Based on field performance, *Dendrocalamus asper*, *D. membranaceus, Bambusa vulgaris*, and *B. blumeana* were identified as promising species for restoring chromium contaminated sites, although continuous monitoring of toxic metals is essential, and further studies involving a broader range of bamboo species are recommended to optimize phytoremediation outcomes (Were et al., 2017).

One major research gap lies in the selection and improvement of bamboo species with stronger translocation and bioconcentration abilities. Most currently studied species exhibit limited metal movement form roots to shoots, which restricts overall remediation efficiency. Future efforts integrating plant breeding, assisted selection, and molecular tools may enable the development of fast growing, high biomass bamboo varieties with enhanced chromium tolerance and accumulation potential (Ranieri et al., 2020). At the same time, deeper insight into plant–microbe interactions within the bamboo rhizosphere is needed. Beneficial associations with chromium-reducing bacteria and fungi can play a crucial role in metal immobilization and detoxification, yet these interactions remain underexplored in Cr(VI)-contaminated environments (Zhang et al., 2020b).

From a materials and engineering perspective, future research should emphasize the development of low-energy, scalable, and cost-effective methods for producing bamboo-derived biochar, hydrochar, and activated carbon. Alternative approaches such as hydrothermal carbonization and environmentally friendly surface modifications show strong potential to reduce energy demands while maintaining high adsorption performance (Chaturvedi et al., 2023; Lou et al., 2023; Odega et al., 2023). Equally important is the optimization of regeneration and reuse strategies for bamboo-based adsorbents, which is necessary to limit secondary waste generation and reduce lifecycle environmental impacts. Finally, integrating bamboo-based phytoremediation with engineered treatment systems—such as constructed wetlands, permeable reactive barriers, and hybrid adsorption–phytoremediation setups—offers a realistic pathway for large-scale remediation of Cr(VI)-contaminated soil–water systems. Such integrated approaches can improve remediation efficiency while simultaneously supporting ecosystem recovery and long-term environmental sustainability. Addressing these challenges in a systematic manner will be critical for advancing bamboo-based remediation from controlled experimental studies to reliable, field-deployable solutions.

## 8 Conclusion

Cr(VI) is a toxic pollutant that contaminates both the biotic as well as abiotic components of the environment, thereby spreading toxicity. Remediation of the same using bamboo as well as bamboo-based derivatives provides better alternatives to other conventional methods. The large biomass and fast-growing nature of bamboo make it an excellent candidate for phytoremediation of soil and water bodies contaminated with Cr(VI). Moreover, the deep dense root network helps them to immobilize heavy metals like Cr(VI) in the soil, thus preventing further contamination. Several bamboo-based derivatives like activated carbon, charcoal, hydrochar etc, provide excellent adsorption properties and are being actively used in remediation purposes. However, there are several challenges faced in this context that need considerable research efforts. More focus may be put on bamboo-based composite materials that are quite promising in the remediation of Cr(VI). Efforts must be made to make such adsorbents eco-friendly and recyclable without causing further secondary pollution.
