# Supplementary material for: Sustainable strategies for remediation of Cr(VI) contaminated soil and water bodies: exploring the potential of invasive bamboo and its derivatives
Source: Front Plant Sci. 2026 Mar 23;17:1753291. doi: 10.3389/fpls.2026.1753291 (PMC13050963; doi:10.3389/fpls.2026.1753291)
Supplement: Supplementary file 1 [file DataSheet1.docx]

**Supplementary Information**

**Table S1.** Conventional remediation approaches for managing Cr(VI) Contamination

| **Remediation Method** | **Key Features** | **Limitations** | **Ref.** |
| --- | --- | --- | --- |
| Chemical reduction and precipitation | Effective conversion of Cr(VI) to Cr(III) using agents like FeSO₄ and Na₂S₂O₅ | Generates sludge, incurs chemical costs, and is sensitive to pH levels | (Liu et al., 2012; Zhao et al., 2018) |
| Adsorption | High removal efficiency of Cr(VI) using activated carbon or resins | Rapid saturation of adsorbents and costly regeneration processes | (Liu et al., 2012; Rahman et al., 2024) |
| Electrochemical methods | Offers fast kinetics and precise control over the remediation process | High energy requirements can limit feasibility | (Liu et al., 2012; Rahman et al., 2024) |
| Microbial bioremediation | Eco-friendly approach with potential for in situ application | Sensitive to environmental factors, which can affect microbial activity | (Peng et al., 2017) |
| Phytoremediation | Utilizes plants like bamboo for root uptake and rhizofiltration, making it low-cost and suitable for large areas | Slow process and challenges in biomass management | (Liu et al., 2012) |
| Biochar Sorption | Involves adsorption and redox reactions, enhancing Cr removal efficiency | Cost varies based on the modification of biochar | (Ahmad et al., 2024) |

**Table S2**. Predominant Cr species at pH 5 – 9 in the aquatic environment

| **Forms of Cr** | **Predominant species at pH (5 – 9)** |
| --- | --- |
| Cr(III) | Cr(OH)_3_, |
| Cr(VI) | HCrO_4_^-^, CrO_4_^2-^ |

**Table S3.** Microbes facilitating Cr(VI) Reduction

| **Condition** | **Microorganism** | **Ref.** |
| --- | --- | --- |
| Aerobic | *Bacillus megaterium* Cr02 | (Liu et al., 2025) |
|  | Bacillus sp. CRB-1 | (Zhu et al., 2019) |
|  | Bacillus and Microbaterium spp. | (Molokwane et al., 2008) |
| Anaerobic | Clostridia sp. | (Khan et al., 2025) |
|  | *Exiguobacterium* sp. PY 14 | (Huang et al., 2023) |
|  | Gammaproteobateria and Alphaproteobacteria | (Khan et al., 2025) |


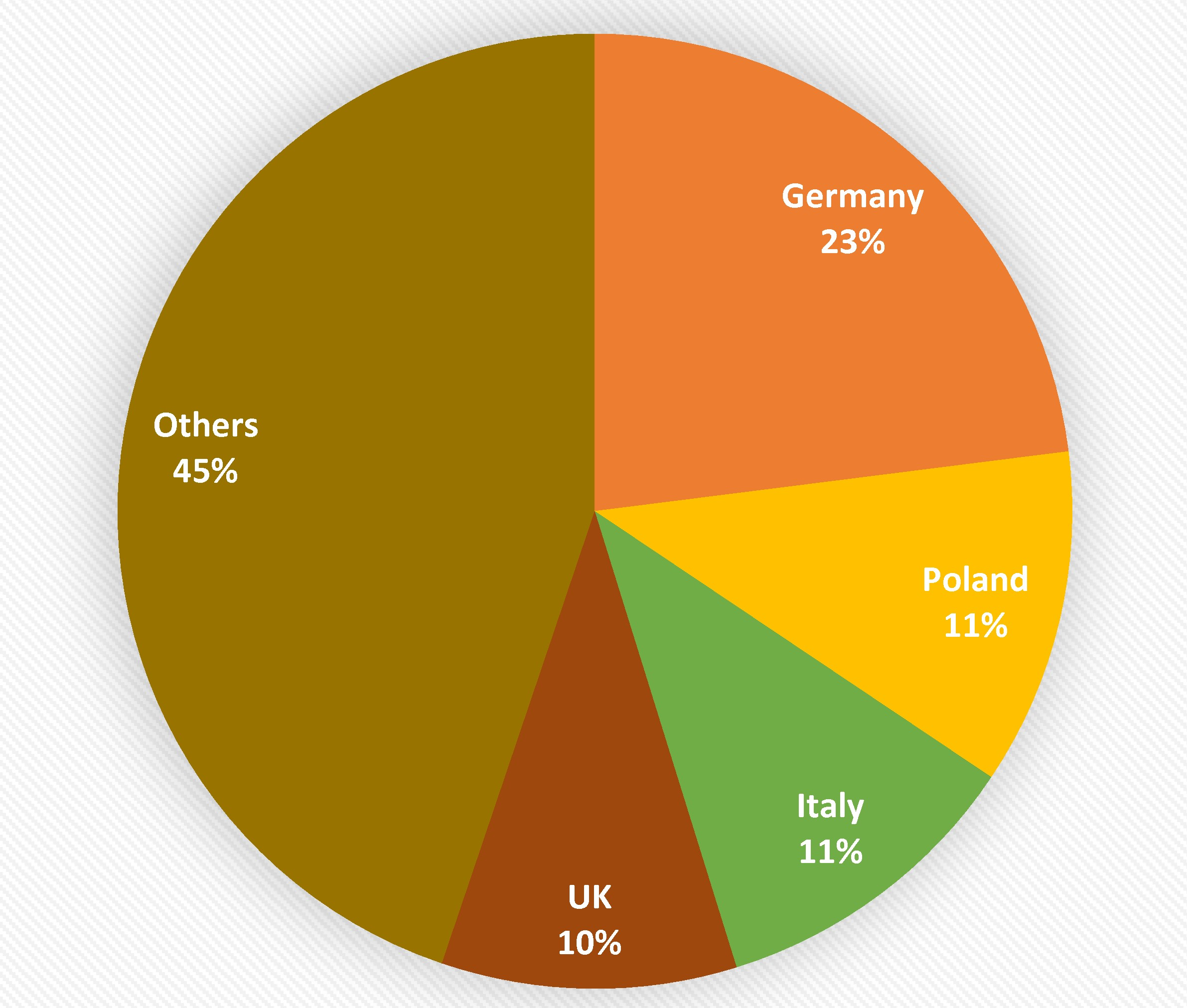


**Figure S1**. Major chromium emitters among the EU in 2017.

**Table S4**. Different current physical and chemical approaches for remediation of Cr(VI)

| **Material Used** | **Remediation Method** | **Efficiency** | **Ref.** |
| --- | --- | --- | --- |
| Phosphorylated chitosan impregnated ethyl cetyl dimethyl ammonium bromide | Physical | Adsorption capacity = 266.67 mg g^-1^.  94% total Cr recovery | (Kahu et al., 2016) |
| Seed pods of lotus | Physical | Absorption capacity = 153.85 mg g^-1^ | (Shi et al., 2020) |
| Polypyrrole-sugarcane bagasse composite | Physical | Adsorption capacity = 156.00–251.00 mg g^-1^. | (Chen and Pan, 2021) |
| Nanoscale metal encapsulated in vanadium-coated chitosan matrix | Physical | Reduction of 92.43% –96.95% Cr(VI) | (Mahmoud et al., 2021) |
| Pyrite | Chemical | 100% removal within 60 minutes | (Li et al., 2020) |
| Acidic solution of Sodium alginate | Chemical | Electrochemical reduction of Cr(VI) to Cr(III) deploying gold electrode | (Butter et al., 2021) |
